# Global properties of regulatory sequences are predicted by transcription factor recognition mechanisms

**DOI:** 10.1186/s13059-021-02503-y

**Published:** 2021-10-07

**Authors:** Zain M. Patel, Timothy R. Hughes

**Affiliations:** Donnelly Centre for Cellular and Biomolecular Research and Department of Molecular Genetics, University of Toronto, Toronto, ON M5S 3E1 Canada

**Keywords:** Regulatory elements, Evolution, Transcription, Bioinformatics, DNA

## Abstract

**Background:**

Mammalian genomes contain millions of putative regulatory sequences, which are delineated by binding of multiple transcription factors. The degree to which spacing and orientation constraints among transcription factor binding sites contribute to the recognition and identity of regulatory sequence is an unresolved but important question that impacts our understanding of genome function and evolution. Global mechanisms that underlie phenomena including the size of regulatory sequences, their uniqueness, and their evolutionary turnover remain poorly described.

**Results:**

Here, we ask whether models incorporating different degrees of spacing and orientation constraints among transcription factor binding sites are broadly consistent with several global properties of regulatory sequence. These properties include length, sequence diversity, turnover rate, and dominance of specific TFs in regulatory site identity and cell type specification. Models with and without spacing and orientation constraints are generally consistent with all observed properties of regulatory sequence, and with regulatory sequences being fundamentally small (~ 1 nucleosome). Uniqueness of regulatory regions and their rapid evolutionary turnover are expected under all models examined. An intriguing issue we identify is that the complexity of eukaryotic regulatory sites must scale with the number of active transcription factors, in order to accomplish observed specificity.

**Conclusions:**

Models of transcription factor binding with or without spacing and orientation constraints predict that regulatory sequences should be fundamentally short, unique, and turn over rapidly. We posit that the existence of master regulators may be, in part, a consequence of evolutionary pressure to limit the complexity and increase evolvability of regulatory sites.

**Supplementary Information:**

The online version contains supplementary material available at 10.1186/s13059-021-02503-y.

## Introduction

Understanding how regulatory sequence operates is central to understanding the function and evolution of genomes. Several lines of evidence indicate that most of the functional DNA in human and other vertebrates is regulatory sequence, rather than part of coding transcripts. Sequence conservation consistently detects > 5% of the genome under constraint (vs. < 2% which is exons), which can be segmented into well over a million discrete elements (vs. < 300,000 exons) [1, 2]. Large-scale surveys for DNase-I hypersensitivity (DHS) and histone marks have yielded similar numbers of elements (3.5 million DHS sites and 2.3 million enhancers, respectively) [3, 4]. These elements are often active only in specific tissues and cell types (typically 100,000–200,000 in any given sample), complicating functional tests. Nonetheless, a substantial fraction validates using reporter assays [5–7], indicating that, to a first approximation, these are reasonable estimates for the number of discrete regulatory sequences. (Note that, throughout, we refer to “regulatory sequence” rather than “promoter” or “enhancer”, because there are many functional similarities between the two [8]).

Dissecting the sequence features that determine the identity and activity of regulatory sequences is a long-standing enterprise in molecular biology (reviewed in [9–12]), and to our knowledge, there is no clear unifying outcome. The fundamental challenge is that individual transcription factors (TFs) lack the sequence specificity needed for the task, typically falling short by orders of magnitude [13, 14]. A key conceptual issue is whether specificity is increased mainly via constraints on spacing and orientation of TF binding sites (TFBSs) (i.e., the “enhanceosome” model, which we refer to as “SAOC”, for “Spacing and Orientation Constraint”), or whether only the collection of binding sites is critical, and not their exact relative positions (the “billboard” model) [9]. These models represent extremes; a spectrum encompassing aspects of both could also be expected.

One way to explore sequence properties that dictate regulatory sequence function is to develop and examine computational models. Computer programs that use sequences as inputs and predict class memberships of the sequences (for example, regulatory vs. non-regulatory) can be built to incorporate properties such as spacing and orientation of TFBSs, often using DNA words (k-mers) as a substitute. Computational models with and without SAOC have been successful at classifying regulatory vs non-regulatory sites (e.g., enhancer vs. non-enhancer) much better than random guessing, but typically not at the level of specificity that cells achieve: algorithms are often 90% accurate [15, 16], but cells are > 98% accurate (i.e., only 1–2% of the genome is an active regulatory site in a given cell type) [3, 4, 17–19].

It is not yet clear what information is missing from these models that would make them more accurate, although it is known that motifs are lacking for many human TFs [20], and that the range of potential multimeric motifs (i.e., intrinsic SAOC among TFs) is almost completely unexplored [21]. SELEX analysis of cooperative binding of randomly chosen pairs of TFs found that between 3 and 9% of all TF pairs displayed preferred spacing and orientation [21], raising the possibility that enhanceosome-like architectures might also arise at random much more often than appreciated. Because the number of TFs is very high (~ 1600 in human) [20], the number of possible pairs and higher-order combinations is extremely large.

Several additional observations are relevant to a global understanding of regulatory sequence. *First*, to our knowledge, there is no widely accepted default length for regulatory elements [22, 23]: whether based on literature examples, conservation, DNAse hypersensitivity, histone marks, and Massively Parallel Reporter Array data, regulatory elements can range from ~ 10 to 1000 bp. Evidence from Drosophila suggests that enhancer size is correlated with the complexity of the process it regulates [24]. *Second*, unlike protein coding genes, which typically fall into families that arise by duplication and divergence, regulatory sites are typically not related to any other sequences in the same genome, as initially illustrated by comparisons among highly conserved non-exonic sequences [25]. *Third*, there is relatively rapid turnover of distal regulatory elements (i.e., enhancers), relative to genes [10, 26]. As gauged by H3K27ac occupancy across the genome, only ~ 40% of liver enhancers are shared between human and macaque, despite these closely related genomes having a neutral nucleotide substitution rate of only 6% [1, 26]. Individual well-studied functional TF binding sites are also often species-specific [27]. Because these species-specific regulatory sites typically arise de novo, these phenomena suggest that there is a high probability of generating functional regulatory sequence by random mutation, and that there are many ways to build regulatory sequences — possibly, many more than the number of regulatory sites in a given genome. This notion is supported by laboratory experiments in yeast [28] and bacteria [29], in which completely random sequences promote transcription at surprisingly high frequency, as well as by simulations of metazoan enhancers [30]. *Fourth*, the long-standing observation that a relatively small number of TFs act as selector genes, master regulators, or pioneers that determine any given cell type and/or have the ability to specify the locations of regulatory sites [31] must be accounted for: the sequence preferences of these proteins would be expected to bear some relation to the locations of active regulatory sites.

None of these phenomena inherently reveal the relative contributions of SAOC to regulatory site identity, but they do present data with which to compare and evaluate models for consistency with observation. Here, we describe a series of such analyses.

## Results

**Fig. 1:**
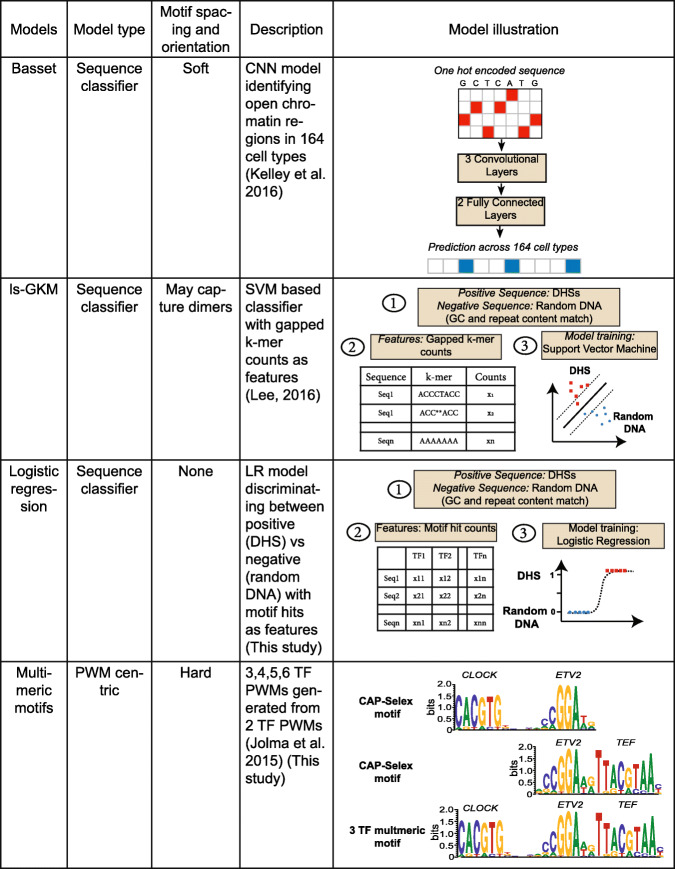
Regulatory element models examined in this study

Our overall strategy was to examine how four different *models* of regulatory sequence detection relate to five different *properties* of regulatory sequences. The four models (Fig. 1) encompassed two established classifiers. “Gapped k-mer” (gkm)-SVM [15] conceptually implements a model without SAOC, because gapped k-mers approximate TF binding sites (or potentially dimeric binding sites), and the feature vector is composed only of gapped k-mer counts per sequence. Basset [16], in contrast, employs a convolutional neural network (CNN) in which experimentally identified regulatory sequences are “one hot encoded” as inputs, following which the first convolutional layer is formed by scanning position weight matrix (PWM) like filters that mimic TF motifs across the sequence input. The subsequent convolutional layers utilize the patterns detected in the previous layers to learn distance and orientation features between them. Basset can therefore learn SAOCs, if they are present in the training data.

**Fig. 2:**
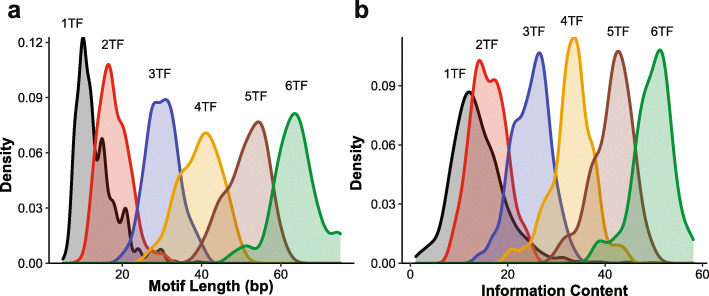
Length and information content of motif collections. **a** Length distribution of monomeric, dimeric, and multimeric motifs. Monomeric and dimeric motifs were experimentally generated and obtained from CIS-BP/HumanTFs and Jolma et al. [21], respectively. Multimeric motifs were obtained from merging dimeric motifs (see “Methods”). **b** Information content distribution of monomeric, dimeric and multimeric motifs

We also implemented two additional models that more explicitly represent extremes. To represent complete absence of spacing and orientation constraints among TFBSs, we trained logistic regression (LR) models with known PWM “hit counts” as features. And, to represent strict spacing and orientation, we extrapolated multimeric motifs from the dimeric motifs of [21], i.e., combining A-B and B-C motifs into an A-B-C motif (as well as 4, 5, and 6 TF combinations) (multimeric PFMs provided in Additional file 1: Table S1). Figure 2B and C show length and information content (IC) calculations for our motif collection. We note that these “multimeric motifs” represent only a very small proportion of all such possible motifs and are therefore not useful as a classifier, but these motifs do have a number of attributes that can be evaluated relative to properties of regulatory sequence.

The regulatory sequence properties (Table 1) included length, frequency, diversity, turnover, and the role of master regulators in cell type definition. Specific data types used as standards differed according to the models and properties being examined, as described below, although overall we present DNase and ChIP data in the embryonic stem cell-H1 (ESC-H1) cell line from the Roadmap Epigenome and ENCODE projects [4, 32]. A sampling of analyses in another cell type (HepG2) yielded similar results.

**Table 1:** Enhancer characteristics examined in this study

Enhancer property	Dataset employed	Tests
Length	• DHS sites from ESC-H1 and HepG2 cell line obtained from the Roadmap Epigenome project • Bigwig files of DHS, H3K27ac, and 5 different TFs from ENCODE project	• ML models with different sized DHS inputs • Metagene plots of DHS and ChIP data • Motif enrichment in DHS data • Examining multimeric motif lengths and IC
Uniqueness/diversity	• N/A	• Review + exploration of enhancer uniqueness from a billboard and enhanceosome perspective
Frequency	• Hg19 human genome	• Score each base in a 500kbp sample using the 3 trained ML models to estimate discriminant threshold for 1% regulatory rate
Turnover	• DHS sites from ESC-H1 cell line obtained from the Roadmap Epigenome project • Enhancer conservation estimates from various studies	• Use ML models to simulate dropout by mutating sequences at neutral mutation rates between species • Capture hits of multimeric motifs in genome, mutate hits at neutral rates and measure dropout after re-scanning
Dominance of master regulators	• Motif collection • Trained ML models	• Train ML models using subset of TFs • Explore learned patterns in ls-GKM models using gkmExplain • Examine LR feature weights using multiple feature selection methods • Poisson estimates of the number of TFs required to specify regulatory sites with and without master regulators • Multimeric motif hits estimation with and without master regulators

We began by considering length and frequency, because these are also parameters that influence the remaining analyses.

### Models are consistent with nucleosome-sized regulatory element lengths

**Fig. 3:**
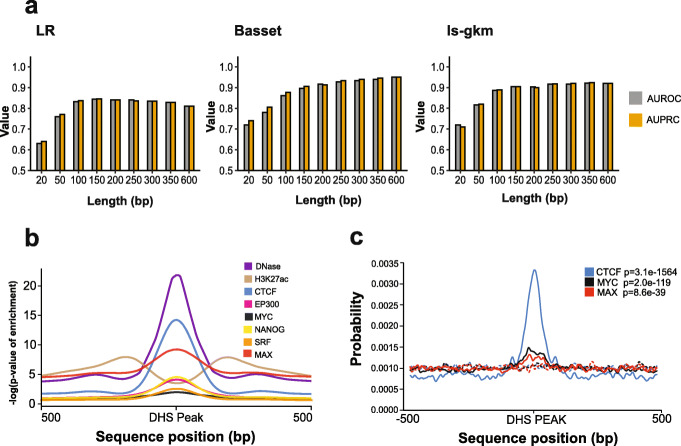
Exploration of regulatory element length. **a** Three ML models (LR, Basset, and ls-gkm) were trained (except Basset) and tested on various DHS lengths as input. Positives were DHS sites obtained from the ESC-H1 cell line. Negatives were random DNA regions of equal length as the positive and matched for GC and repeat element content. **b** Metagene plots for DHS, H3K27ac, EP300, and four TFs in the ESC-H1 cell line. Read signals (negative log *p*-value of signal over control) were averaged at bp resolution (using data from bigwig files) across the regions using DHS peak as reference. **c** Motif enrichment results using Centrimo. Dotted lines correspond to dinucleotide shuffled positives

We began by examining how the three classifiers performed at identifying regulatory sites using identical loci but with sequences extended to different lengths. In Fig. 3A, results are shown for DHS sites from the Roadmap Epigenome project in the ESC H1 cell line [4], with randomly selected genomic regions with matching GC and repeat content as negatives. Outcomes were similar overall when tested on other sample types and with other negatives (e.g., dinucleotide shuffles) (data not shown). LR and gkm-SVM were trained on input sequences ranging from 20 to 600 bp. Basset, which requires 600 bp sequences for training and testing, was given sequences with shuffled flanks extended to 600 bp (see “Methods”).

Strikingly, all three classifiers achieved maximal or near-maximal performance at around 200 bps (Fig. 3A), the approximate length of a nucleosome footprint and flanking linker sequence. Similar results were obtained in the HepG2 cell type as well (Additional file 2: Figure S1A). DHS reads and TF-ChIP enrichment are also largely found within a region of this length, while H3K27ac in flanking regions is consistent with the presence of nucleosomes (Fig. 3B). Motifs for CTCF, SOX2, and MAX [33] are also enriched in the central region the DHS peaks, but not flanks (Fig. 3C), also consistent with regions of this size containing sequence cues involved in delineation of the regulatory sites.

We also found that even the longest multimeric motifs were generally shorter than 100 bp (Fig. 2A). We note that the minimal IFN-β enhanceosome DNA sequence is only 55 bases [34], encompassing binding sites for 8 different TFs. Thus, while the multimeric motifs cannot be evaluated here in a predictive format, their length is consistent with regulatory elements typically having a nucleating sequence of fewer than 100 bases.

These observations indicate that, on a genomic scale, the essential components of regulatory sites are relatively small. Several recent papers employing diverse laboratory assays support this notion [6, 8]. For the remainder of the analyses presented here, we used a length of 200 bases where possible.

### Models are consistent with regulatory element uniqueness

Most regulatory sequences in a genome are unique, i.e., they contain no significant similarity to each other, and aside from the fact that many are found within ancient retroelements, little evidence for origin by duplication. The three classifiers used here are, by design, consistent with the uniqueness of regulatory elements, as is the billboard model itself, since the relative order and spacing of TFBSs is either not used or not required, and the number of possible arrangements of multiple TFBSs within a 200-bp window is astronomical. Thus, uniqueness could be expected from such models, and this prediction is borne out by simulations [30]. We note that gkm-SVM and Basset perform similarly in our tests (Fig. 3A), supporting the notion that little is gained by considering spacing and orientation between motifs in this framework.

The uniqueness of regulatory elements is also an expected consequence of a model in which multimeric motifs with strict SAOC are predominant, provided that the number of distinct combinations of TF motifs exceeds the number of regulatory elements in the genome. Assuming ~ 3% prevalence of cooperative dimeric binding sites among all possible TF pairs, this threshold is exceeded by all motifs comprised of four or more motifs (Table 2).

**Table 2:** Expected occurrences of multimeric motifs in human genome

	1 TF	2 TF	3 TF	4 TF	5 TF	6 TF
Possible combinations	1600	1.3 × 10^6^	2.1 × 10^9^	3.3 × 10^12^	5.2 × 10^15^	8.4 × 10^18^
Percent that cooperate (%)	100	3	0.09	0.0027	8.1 × 10^−5^	2.4 × 10^−6^
Expected number of motifs	1600	38000	1.8 × 10^6^	8.8 × 10^7^	4.3 × 10^9^	2.0 × 10^11^
Effective motif length (based on IC)	6	8	12.25	16.25	21	25.5
Individual motif hits in human genome	7.3 × 10^5^	4.6 × 10^4^	1.3 × 10^2^	**0.49**	**0.00068**	1.3 × 10^−6^
Total motif hits in human genome	1.2 × 10^9^	1.8 × 10^9^	2.3 × 10^8^	**4.4 × 10**^**7**^	**2.9 × 10**^**6**^	**2.8 × 10**^**5**^

Columns indicate number of TFs in multimeric motifs

Thus, the uniqueness of regulatory sequences is consistent with any of the models considered here, but it places significant constraints on a model in which spacing and orientation of TFBSs are dominant: four or more TFs are required, if assumptions are roughly correct. We note that this figure is compatible with the requirement for five cooperating TFs obtained above when considering the frequency of regulatory sequences.

### Models are compatible with observed frequency of regulatory sites

Regulatory sites are relatively rare, as discussed above. In a typical human cell type, 1–2% of the genome displays hallmarks of regulatory activity (e.g., DHS sites, H3K27ac) (100–200,000 individual sites), while evolutionary constraint and aggregate biochemical data indicate that 5–10% of the genome may be regulatory sequence (totaling 1–3 million distinct sites) [1, 3, 4].

**Fig. 4:**
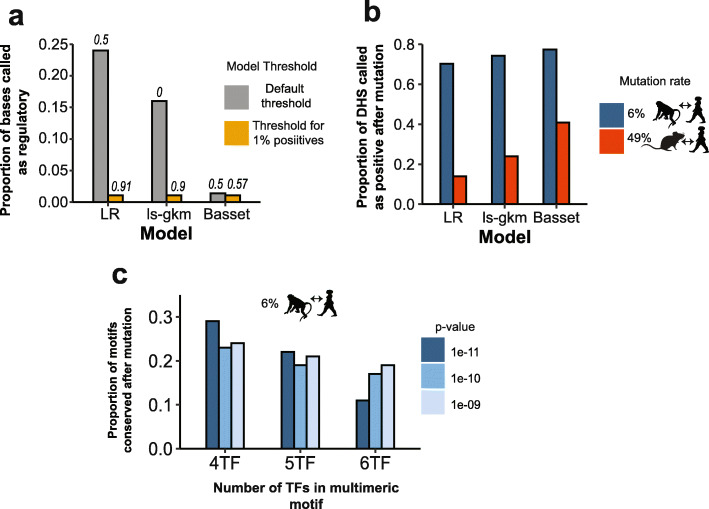
Turnover of regulatory regions based on simulated mutations at neutral rate. **a** Proportion of bases identified as regulatory by the three models based on DHS data in the ESC-H1 cell line. Gray represents the intrinsic model threshold (in italics), whereas the yellow bars represent the modified threshold to obtain ~ 1% positive rate. **b** Proportion of DHSs called as positives (based on scoring the test set using the models) after simulated mutations at the neutral rate between human/macaque (6%) and human/mouse (49%). **c** Proportion of multimeric motif hits (at *p*-values corresponding to unique hits) conserved in the axolotl genome (32 Gb) after simulated mutations at the neutral rates between human/macaque and human/mouse. Silhouettes were obtained from PhyloPic

The classifiers we examined are all trained on balanced data (i.e., an equal proportion of positives and negatives) and employ an intrinsic discriminant value that is optimized for balanced data but can be modulated. In all cases, the intrinsic discriminant value threshold classifies a large proportion of the genome as positive (Fig. 4A), presumably because a small false-positive rate has much less impact in the training than it does in the testing scenario. The number of elements and proportion of sequence detected by classifiers as “positives” can be modulated, however, by raising or lowering the discriminant value employed. This aspect makes these models compatible with the observed frequency of regulatory sites. Discriminant values that yield the expected proportions are given in Fig. 4A. These values yield a larger number of false-negatives but are used in analyses below because they are more realistic.

For the multimeric motifs, the number of expected occurrences can be extrapolated over a human genome-sized sequence, using the IC as a guide, i.e., assuming that the best binding sequences are those that are employed to delineate regulatory sequences (Table 2) and that the number of cooperative TF pair combinations is similar to the 3% described in Jolma et al. In these calculations, the expected number of matches to 5-TF motifs is similar to the expected total number of regulatory sites (~ 3 million), while the number of 6-TF motifs that would have a strong match in the genome is too small to occur at random, despite their large numbers, suggesting that this level of complexity would occur by adaptation and not random processes.

This result is only an approximation; Jolma et al. [21] noted that multiple spacings for the same TF pair are frequent and that the CAP-SELEX assay employed has false-negatives; in addition, it is known that not all functional TFBSs are high-affinity [35]. In addition, the calculations in Table 2 assume that all possible combinations of 5 TFs are present in some cell type. If only a subset of all possible combinations of 5 TFs are ever present at the same time, then the number of total motif hits that would ever be active in the genome would decrease. For example, we estimate 43 million motif hits for all possible 4 TF combinations, but if only 5% of all four TF combinations ever have all four TFs present and active in the same cell type, then only ~ 2 million motifs would ever be active in the genome — roughly consistent with estimates for the total number of expected regulatory sequences.

Thus, all of the models we considered are compatible with the observed frequency of regulatory sites. The classification algorithms may benefit from modification to address the imbalance in positives and negatives. If multimeric motifs are employed broadly, these analyses indicate that they will involve 4–5 TFs.

### Models are consistent with regulatory element turnover

Regulatory sequences turn over much more quickly than genes. Published figures for human-macaque span from 11% gained since the common ancestor for developing limb [36] (and ~ 66% conservation between the two, assuming both gain and loss in both lineages) and ~ 40% conservation between the two for liver [26]. Figures for mouse-human include 30% conservation for predicted regulatory DHSs [37] and 20% conservation of H3K27ac signals for liver [26].

We asked how the computational models react to mutation of human sequences that score as positives or negatives by the models. We employed the ESC-H1 DHSs (as above) for this analysis, because the ChIP-defined enhancers tend to be large, and it is unknown which sequences within them are important for enhancer identity. We considered two evolutionary distances (human-macaque and human-mouse). In these analyses, we randomly mutated the DHS sites at the neutral rates (6 and 49%, respectively) [1] and then re-scored the sequences, asking if they fell below the discriminant values (determined above for 1% positivity). We used the neutral rate because the vast majority of bases within measured regulatory elements appear unconstrained [26]. The simulated dropout rates varied somewhat but, for all three models, were roughly similar to the ranges that have been reported in the literature (we obtained 70–80% conservation for macaque and 15–40% for mouse) (Fig. 4B).

To examine the effects of mutations on SAOC, we identified sequences that have high motif scores for our multimeric motifs from among a very large amount of non-human DNA sequence, in order to obtain a large number of motif hits (by the reasoning above, each motif should have only one strong match in the human genome, resulting in very few examples). We note that the motif score cutoffs reflect the frequency at which motif matches arise in random sequence; thus, by default, such sequences will occur at random. As we do not know the relevant physiological score thresholds, in this analysis, we considered loss rates several motif score cutoffs (presumably reflecting binding strength) and different numbers of cooperating TFs. We mutated the motif hits as described above. Strikingly, at the human/macaque substitution rate, the dropout results are roughly similar to the experimental enhancer conservation rate; we obtained 10–30% conservation (vs 40–66% from experiments in the literature). This result is robust to variation in the number of TFs and *P*-value cutoff (Fig. 4C). At the human/mouse substitution rates, however, the simulation yielded a much greater loss of multimeric enhancers than what is observed in experimental data (zero enhancers left).

Thus, the observed turnover in enhancers is expected for sequences evolving near the neutral substitution rate, regardless of whether the model of the sequence recognition mechanism encompasses SAOC. We note that these analyses do not incorporate complexities such as evolution of regulatory sites to rely on different TFs over longer timescales. Such a mechanism could explain why the multimeric motifs predict near-zero conservation at human/mouse divergence, even though many conserved enhancers do exist at this distance [26].

### Models are consistent with a complex regulatory environment that includes master regulators

**Fig. 5:**
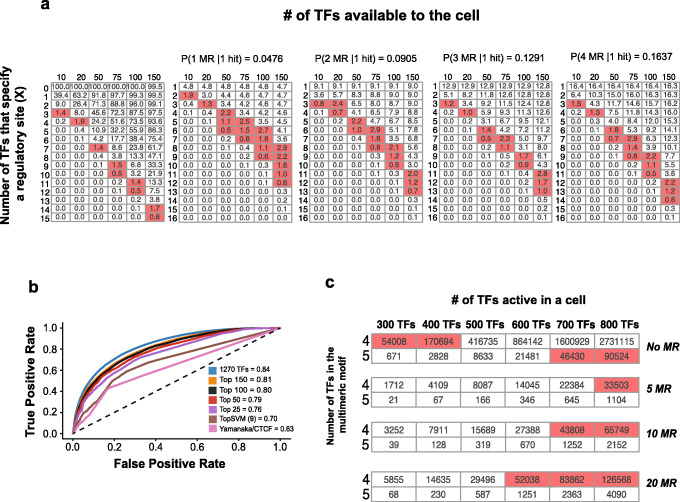
Exploration of master regulator TFs. **a** Leftmost heatmap indicates the cumulative probability of > *X* hits (probability of a hit is 1/20 in a 200-bp region) calculated as a Poisson distribution where the parameters are dependent on the number of active TFs in a cell and the numbers of TFs that specify a regulatory site. Remaining heatmaps show the effect of master regulators in the same framework in which probability in the leftmost heatmap is multiplied by the probability of getting a single master regulator (MR) hit [Poisson(1 MR hit | 1–4 MR available to the cell]. Cells with red shading are close to the expected probability of active regulatory elements in a cell type (i.e., ~ 0.5–3% of the genome). **b** AUROC curves for the trained LR models on DHS data in the ESC-H1 cell line using subset of the 1270 TF motifs identified using recursive feature selection (top 25, 50, 100, and 150 motifs). Models were also trained on motifs identified by gkm-Explain/TF-Modisco (9 motifs) and just the Yamanaka factors + CTCF (5 motifs). **c** Heatmaps indicating the number of 4-TF and 5-TF multimeric motif hits in the human genome, based on 3% cooperativity rate of dimers and the presence (5, 10, 20 MR) and absence of MR. Cells containing values between 30,000 and 200,000 are shaded red corresponding to the typical number of active regulatory elements in a cell type

We used several approaches to ask how the models reflect the existence of master regulators. We began by considering theoretical arguments regarding the number of TFBSs and combinations thereof that would be required to delineate a regulatory sequence, in the absence of SAOCs. Wunderlich and Mirny [14] have made similar calculations, assuming a Poisson distribution and motif information content to estimate the minimum number of binding sites required in a sequence of a particular length, such that the cluster of binding sites is unique in the genome. The estimates were based on regulatory sequences of size 1000, however, and did not consider a wide range of TF numbers. We revisited the Poisson approach using the simple assumption that each TF specifies six bases (consistent with Fig. 2) and that regulatory sequences are 200 bp long. We also surveyed many different numbers of TFs. Figure 5A shows that many configurations (of TFs contributing to regulatory sequence identity, and TFBSs required per regulatory element) are consistent with the observed 1–2% of the genome functioning as regulatory sequence in a given cell type. The main constraint observed is that if a larger number of TFs *can* function in delineating regulatory sites, then the number *required* to delineate a regulatory site must be larger. For example, with only 10 active TFs, then three TFBSs confer sufficient specificity; for 50 TFs, 7 TFBSs; and for 100 TFs, 11 TFBSs.

We next considered the case in which a regulatory site requires the existence of a binding site for a hypothetical master regulator (which in this sense could also be considered a selector or pioneer TF), in addition to other specifying factors as above. This notion is suggested by the observation that, in ES cells, half of all regulatory sites contain a binding site for at least one of five master regulators (including CTCF), even though none of the master regulators is sufficient to specify regulatory sites independently (Additional file 2: Figure S2). In this analysis, we assumed that the master regulators have the same level of sequence specificity as other TFs. The addition of a requirement for a hypothetical master regulator has the effect of dramatically reducing the number of additional TFs needed to achieve specificity observed in cells (Fig. 5A). This outcome holds even allowing for multiple master regulators, each of which can suffice. Thus, while a collective of multiple TFs would still be needed, the master regulator has the effect of reducing the necessary complexity of regulatory sites, because the number of permissible TF combinations drops.

The numbers of TFs required under plausible scenarios in Fig. 5A are small enough that it should be possible to learn very accurate models, if there are no specific interactions among the TFs: there are orders of magnitude more examples (regulatory sites) than features (TFBSs) (e.g., 100,000 DHSs vs. 100 TFBSs). We therefore asked whether the models could perform well with a small number of TFBSs. The LR model is most easily manipulated in this way. A model using only 150 TF motifs (those with highest weights based on recursive feature selection) performs as well as the full collection of 1270 motifs; smaller models (i.e., < 25 motifs) are greatly compromised, however (Fig. 5B), including a model using only motifs for CTCF and the four Yamanaka factors (Oct4, Sox2, Klf4, and c-Myc) (which are the expected master regulators for ES cells). We did not attempt to identify the most predictive filters in Basset, which was originally reported to utilize hundreds of filters representing hundreds of TF motifs [16]. But, ls-GKM models can be readily interpreted using gkm-Explain [38] coupled with TF-Modisco (https://arxiv.org/abs/1811.00416) to obtain predictive motifs, which we reasoned may be superior to cataloged motifs. This process identified a relatively small number of consolidated motifs (5–9, depending on data selection), but these motifs were not highly predictive when we used LR to learn models with the motifs (Fig. 5B).

**Fig. 6:**
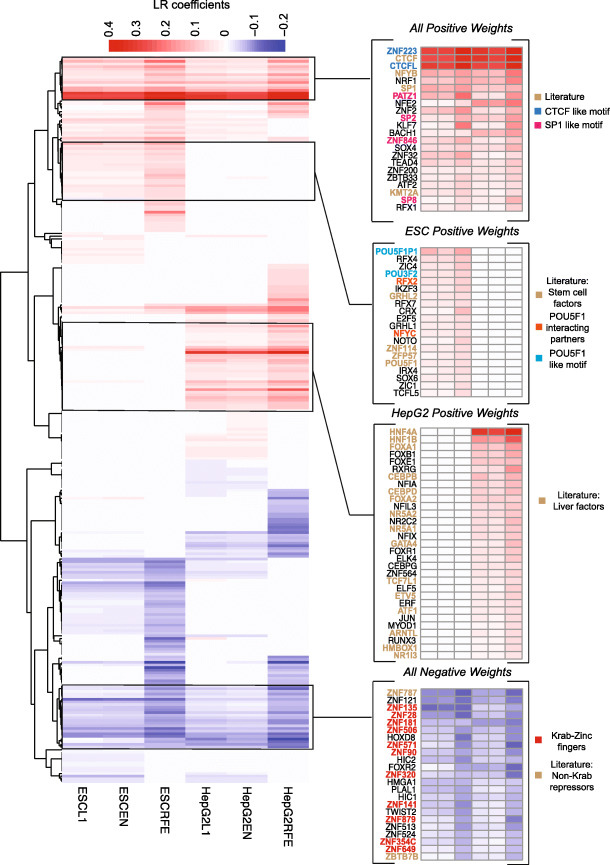
Exploration of LR feature weights. Heatmap of feature weights learned by the LR model in two different cell types (ESC-H1 and HepG2) using three different feature selection methods (ElasticNet, L1, and RFE) to obtain ~ top 150 features each. Rows are clustered using hierarchical clustering with uncentered Pearson correlation as the similarity measure and average linkage. Pullouts show four groups of features with the indicated characteristics

To ask what insight could be gained from these models, and whether they are reproducible and consistent with existing knowledge, we examined the features retained in the 150 TF model. We also considered alternative feature reduction methods (ElasticNet and Lasso regression) and another cell type (HepG2). The resulting feature weights for the six models (3 feature selection methods x 2 cell types) are shown in Fig. 6 (Heatmap with all TF names shown in Additional file 2: Figure S3). Different feature reduction methods yielded similar overall models for the same cell type, with similar predictive values (Additional file 2: Figure S1B). Strikingly, the motif weights strongly reflected known regulatory functions within the corresponding cell types. Features assigned high weights for ES cells included at least six known ES cell regulators, among which were POU5F1 [39], ZFP57 [40], ZNF114 [41], GRHL2 [42], NFYC, and RFX2 [43]. Those with high weights for HepG2 include at least seven TFs with established functions in liver, including HNF4A [44], HNF1B [45], CEBPB [46], FOXA2 [47], NR5A2 [48], GATA4 [49], and NR1I3 [50]. TFs with highest weights in both cell types included known chromatin modulators CTCF [51] and KMT2A [52], promoter-recognition factors Sp1 [53] and NFYB [54], and a panel of TFs with related to those of motifs CTCF (CTCFL and ZNF223) and SP1 (SP2, SP8, PATZ1) (Additional file 2: Figure S4). Intriguingly, 23 motifs are given negative weights in all models; these include 11 KRAB-C2H2 proteins, which are typically repressors [55]. It seems unlikely that such a strong correspondence between LR feature weights and independently derived biological properties of the TFs would have been obtained by coincidence; we take this outcome to indicate that the features weights — and the relatively large number of features retained — are biologically meaningful.

We also produced corresponding estimates of how many TFs would be needed for multimeric motifs to be consistent with observation in a single cell type, and the impact of incorporating of master regulators. We focused on four or five TFs cooperating with SAOC in order to specify each regulatory site, as per the analyses above. We calculated how many such combinations are expected, and how many regulatory sites would be specified per genome, as a function of the number of TFs expressed, assuming all of them can contribute to regulatory site formation (Fig. 5C). These calculations were then repeated assuming a small number of master regulators (5, 10, or 20) that are obligatory for a regulatory element to function. In this regime, with no master regulators, the number of TFs required to specify the observed number of distinct active regulatory sites per cell type (100,000–200,000) is ~ 300 (for 4 TF motifs) and ~ 800 (for 5 TF motifs). But, if a regulatory site must contain a binding site for at least one of a small number of master regulators, then the number of working combinations of TFs drops precipitously, to a point that most scenarios examined are not feasible. The only workable regimes are those with multimers of 4 TFs, and 700–800 contributing TFs beyond the master regulators. These constraints loosen as the number of master regulators increases (approaching those of the model that does not consider master regulators).

Collectively, these outcomes support the notion that regulatory site identity is specified by a relatively large number of factors. The LR models described above do rely most heavily on a relatively small subset of TF motifs, consistent with the notion of master regulators. But, together with the estimates above, they also suggest that many TFs contribute to regulatory site identity, even if they do not significantly influence the overall state of the cell. We also conclude that the existence of master regulators does not exclude models with or without SAOCs, but it does present significant constraints for both models — most dramatically in the case of strict multimeric motifs.

## Discussion and conclusions

Overall, models of sequence recognition both with and without SAOC are broadly consistent with all properties of regulatory sequence examined here. Thus, we cannot exclude either model. Moreover, while some of the global properties of regulatory sequence appeared striking when first reported and are still subjects of active study (e.g., turnover rates), they are in fact expected, given conventional wisdom about how gene regulation works. The analyses do identify apparent constraints and considerations, however, mainly with regard to the number of TFs that are likely to be required for regulatory site specification in a human-sized genome. For a model with full SAOC, in the framework described here, only 4- and 5-TF multimeric binding sites yielded feasible numbers, and only 4-TF sites, coupled with a large number of contributing TFs, are consistent with a model including master regulators across all parameters examined. For complete lack of SAOC, we estimate that hundreds of TFs are likely to contribute to regulatory site identity, even though only a handful of TFBSs (5–15) are required to nucleate an individual regulatory site. These numbers are also consistent with current estimates (e.g., [56]) and, we believe, conventional wisdom (but, to our knowledge, it has not been previously demonstrated in this fashion, nor has it been considered by what regime such a system would operate and evolve). In both cases, however, the existence of master regulators (and the fact that regulatory sites cannot accurately be predicted using only master regulator motifs) suggests that there may be forces that cause these TFs to emerge as dominant, but to still rely on many other factors for their activity.

We emphasize that these analyses are aimed at how regulatory site identity is established, not at understanding all functions of regulatory sites. For example, all of the models we examined are consistent with a fundamental regulatory element size roughly similar to the amount of DNA that would encompass one nucleosome and its flanking linkers (150–200 bases). This outcome is unsurprising given that it is broadly consistent with the appearance of many genomic data types — “peaks” of many types are often this size, on average. To our knowledge, however, the demonstration that all predictors work well with such short sequences is new. The fact that many well-studied enhancers are much larger, and that longer sequences promote higher expression in MPRAs (e.g., [57]) may be explained as modifications of basic regulatory units to perform additional specialized tasks. This notion is consistent with the fact that low-affinity TF binding sites in enhancers contribute to regulatory activity, even though these sequences would not be sufficiently strong to specify the identity of the regulatory sequence on their own [35].

Perhaps the most intriguing issues to emerge from these analyses revolve around the existence of master regulators. It is not surprising that such factors exist — some TFs will have higher abundance than others, or higher binding affinity, or capability to recruit cofactors such as nucleosome remodellers. It is also not surprising that such proteins might still cooperate with other TFs. But how regulatory sites evolve within such a scheme bears further consideration, as does the over-arching question of the evolutionary pressures and constraints that would produce such a system, i.e., what specifically drives specific numbers of master regulators per cell type.

One possibility is that master regulators could both reduce the complexity and increase adaptability of regulatory sites, by having a single dominant TF binding site and many potential associated cofactors that can change. Master regulators may also be selected for their ability to determine cell type identity in a minimally complex fashion. The results of the LR analysis, however — in which dozens of TFs selected by the model can be easily rationalized on the basis of literature knowledge — suggests that the notion of master regulators is oversimplified and that the contribution of TFs to regulatory site identity (and, presumably, cell type identity) is instead on a sliding scale. Gene functions with small effect sizes are often difficult to detect in laboratory experiments, and the same is likely true for the effects of TF binding sites. Additionally, the LR model can be used to identify cell type specific regulators. We suggest that further study of such considerations employing approaches similar to those utilized here may further illuminate the nature and variety of regulatory sequences.

## Methods

### Datasets

For LR, Basset, and ls-gkm, ESC-H1 and HepG2 DHS sites (*n* = ~110,000) were used as positive sequences and the bed files downloaded from the Roadmap Epigenome project and ENCODE, respectively [4, 32] (Additional file 3: Table S2). Positive sequences of varying lengths were obtained by extending from the peak center equally in the 5′ and 3′ directions. We generated negative sequences of equal length, GC content, and repeat element fraction as the positive sets using the genNullSeqs command in the R-package “gkm-SVM” [58].

### ML models

For the LR model, we used 1270 human TF motifs obtained from CIS-BP [33] and HumanTFs [20] (Additional file 4: Table S3). We used the MOODS package [59] (*p* = 1e− 4 and batch settings) to compute motif hit counts (features) in the positive and negative sets. To scale the hits, we used the standard scaler function from the Python Sci-kit learn library (http://jmlr.csail.mit.edu/papers/v12/pedregosa11a.html). To train the LR model, we used the logreg function with default parameters from the Python Sci-kit learn library. For the SVM model we used the ls-GKM package [60]. We used ls-gkm with default settings, i.e., the wgkm kernel along with word length (*l*) = 11 and number of informative columns (*k*) = 7. For Basset, we used the pre-trained model deposited in the Kipoi repository [61] to score the positive and negative sequences.

For the train-test split (for LR), we used five different splits of trained the LR model on data from 16 chromosomes and tested on the remaining 7. For ls-GKM, due to long compute times, we used the same splits but trained on only 6 of the 16 chromosomes, and tested on the same 7. The same test sets were scored using the Basset pre-trained model (which had been exposed to the training sequences, but we note that its performance was only slightly better than ls-GKM). Negatives for Basset scoring of various lengths included sequences with dinucleotide shuffled flanks, using the script fasta-dinucleotide-shuffle-py3.py provided in MEME suite [62] to represent the various lengths examined in this study.

For reduced features sets, we used the recursive feature selection function from Pythons Scikit-learn package to determine the top 25, 50, 100, and 150 features (out of the 1270 TF motifs). LR models were then trained using this subset of features. For feature selection using elasticnet, the following parameters were used in logreg function to obtain ~ 150 features (penalty: elasticnet, solver: saga, *C* = 0.0007 and l1_ratio = 0.5). For feature selection using lasso regression, the following parameters were used in the logreg function to obtain ~ 150 features (penalty: l1, solver: saga, *C* = 0.00125).

To interpret the trained ls-GKM model used gkm-Explain [38] and computed both importance scores and hypothetical importance scores on the positive set using the gkmexplain command (-m 0 for importance scores and -m 1 for hypothetical importance scores). These scores were input into TF-Modisco (https://arxiv.org/abs/1811.00416) to generate PWMs. We used TomTom [63] to identify which TFs the PWMs correspond to in databases described above.

### Metagene plot and motif enrichment

We downloaded bigwig files (*p*-value of signal over enrichment) of DHS regions, EP300, H3K27ac, and 5 TFs (CTCF, MAX, MYC, NANOG, SRF) in the ESC-H1 cell line from the ENCODE project [32] (Additional file 3: Table S2). To determine whether TF motifs were enriched in the center of the DHS sequences, we ran Centrimo [64] (with dinucleotide shuffled negatives) on the same subset of DHS peaks used to generate the metagene plots, using motifs obtained from Jaspar (CTCF: MA0139.1; SRF: MA0083.2; and MAX: MA0058.3) [65].

#### Motif collection and information content calculations

Monomeric motifs were obtained from CIS-BP and HumanTFs as above. Dimeric motifs (*n* = 516) were obtained from [21]. Multimeric motifs (3 or more motifs) were generated by the following procedure. We first manually annotated dimer motifs (A-B, C-B) from Jolma et al. [21] to determine if one or both of the monomeric motifs were palindromic and in what order the TFBSs occurred. We then identified all the possible 3-TF motifs that can be generated accounting for dimers containing a palindromic sequence (3-TF A-B-C motif is only possible if B is palindromic in dimeric motifs A-B and C-B). We then merged dimer motifs into 3-TF motifs by first aligning the two dimers with STAMP (parameters: -cc PCC and -align SWU) [66] to obtain the PFM alignment (manually verifying the correct orientation). At overlapping positions in the alignment, PFM scores in the dimeric motifs were averaged per base, per position to produce the 3-TF motif; scores at non-overlapping positions were unchanged. To generate larger multimeric motifs (4, 5, and 6 TFs) we repeated these steps iteratively by merging the computationally derived 3-TF motifs with dimers to form 4-TF motifs etc. Multimeric motif PWMs are provided in Additional file 1: Table S1. To calculate the information content for all the motifs in our collection, we used the getIC function from the R Package “MotifStack” [67]. Multimeric motif hit calculations in Table 2 and Fig. 5C have been provided in Additional file 5: Table S4.

#### Frequency calculations

To calculate frequency of “positives” for classifiers, we selected a random 500 Kb region of the human genome (hg19: chr20 2,000,000–2,500,000). We divided this region into overlapping 200 bp sequences (600 bp for Basset, but with dinucleotide shuffled 200 bp flanks) and scored using the models (trained on 200 bp DHS data, except Basset, which was pre-trained as noted above). Thresholds modified to 1% positivity were used for turnover calculations.

#### Simulated evolution and turnover calculations

To simulate random evolution and dropout for the classifiers, we mutated positive and negative sequences from our test sets from the 200 bp models (LR, ls-GKM and Basset) (using sequences that are true positives and negatives and that score in the models as positives and negatives, respectively). We randomly mutated these sequences at known neutral rates for human/macaque (6%) and human/mouse (49%) with equal probability for each base change. We then re-scored these sequences using the three models.

To simulate random evolution and dropout of multimeric (i.e., SAOC) regulatory elements, we used the computationally generated multimeric motifs (4, 5, and 6 TF motifs) to scan a large amount of sequence (the 32-Gb axolotl genome [68], for convenience), at three MOODS *p*-value cutoffs (1e^−11^, 1e^−10^,1e^−9^), as described above, in order to identify a large number of motif hits at varying thresholds (i.e., predicted affinity). We then mutated these same sequences at established substitution rates as described above and rescanned using MOODS.

#### Poisson calculations

We estimated the number of TFBSs required to specify a regulatory sequence without SAOC as follows. Given that a DHS region is ~ 200 bp and that the IC a single TF motif is equivalent to ~ 6 bp, the probability of a perfect binding site in a 200-bp region is ~ 1/20, given that a perfect match to a non-palindromic 6-mer occurs every 2 kb. We then estimated the probability of *X* hits using the Poisson.Dist(*X*, Mean, False) function in Excel where the parameters were number of TFs required to specify a regulatory site and the expected mean (i.e., 1/20 × no. of active TFs in a cell type and 1/20 × no. of active master regulators in cell type). The entire calculations are provided in Additional file 5: Table S4.

## Supplementary Information

**Additional file 1 **: **Table S1**. Multimeric motif PFMs.

**Additional file 2 **: **Figures S1-S4**.

**Additional file 3 **: **Table S2**. Exact file IDs and links to all data obtained from ENCODE and Roadmap Epigenome.

**Additional file 4 **: **Table S3**. CisBP IDs and gene names for motifs used in LR Model.

**Additional file 5 **: **Table S4**. Poisson estimates and multimeric motif hits calculations.

**Additional file 6.** Review history.

## Data Availability

Multimeric PWMs are provided in Additional file 1: Table S1. All DNase-Seq, TF, and H3K27ac ChIP-Seq data used for analysis are from the ENCODE and Roadmap Epigenome repositories [4, 32] (further details in Additional file 3: Table S2 and “Methods”). Single TF PWMs were obtained from CIS-BP [33], Human TFs [20], and JASPAR [65] databases. Dimeric PWMs were obtained from Jolma et al. [21].
