# Supplementary material for: Global properties of regulatory sequences are predicted by transcription factor recognition mechanisms
Source: Genome Biol. 2021 Oct 7;22:285. doi: 10.1186/s13059-021-02503-y (PMC8496038; doi:10.1186/s13059-021-02503-y)
Supplement: Supplementary file 2 — Additional file 2 : Figures S1-S4. [file 13059_2021_2503_MOESM2_ESM.pdf]

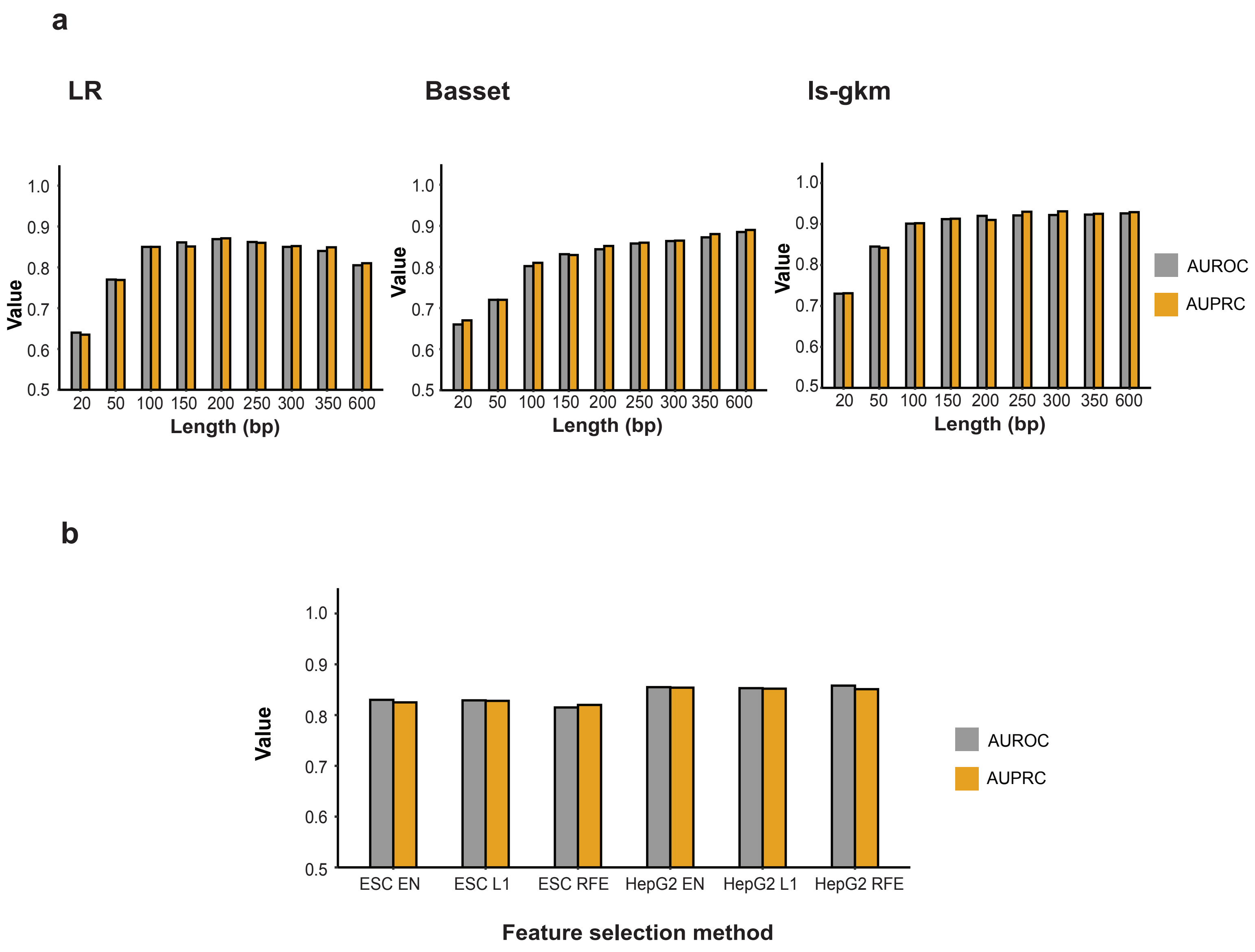

Figure S1: **AUROC/AUPRC values for selected classifiers in this study**. a) Three ML models (LR, Basset and ls-gkm) were trained (except Basset) and tested on various DHS lengths as input. Positives were DHS sites obtained from the HepG2 cell line. Negatives were random DNA regions of equal length as the positive and matched for GC and repeat element content. b) AUROC/AUPRC values of the LR model trained on DHS sites (200 bp) from ESC-H1 and HepG2 cell types using ~ top 150 features (for each cell type) obtained after feature selection via three different methods (ElasticNet, L1 and RFE).

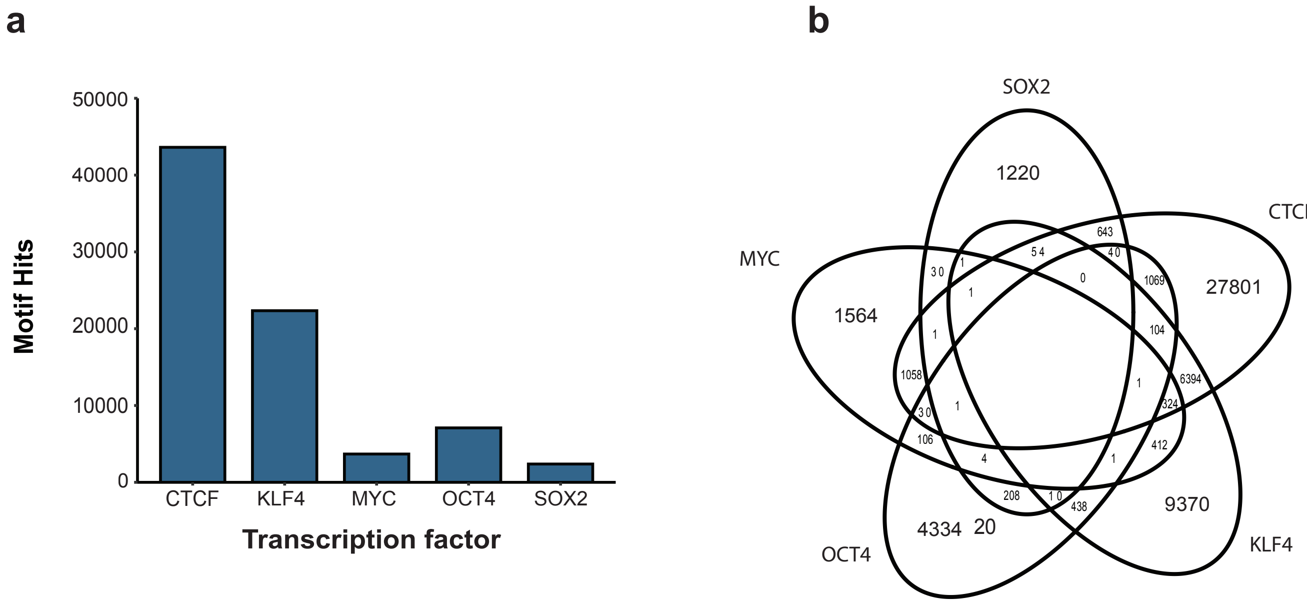

Figure S2: **Motif hits in ESC-H1 DHS sites**. a) Number of motif hits for ESC-H1 regulators (Oct4, Sox2, KLF4, and MYC) and CTCF, within ESC-H1 DHS sites. b) Venn diagram indicating overlap of ESC-H1 DHS sites containing motif hits for ESC-H1 regulators (Oct4, Sox2, KLF4, and MYC) and CTCF.

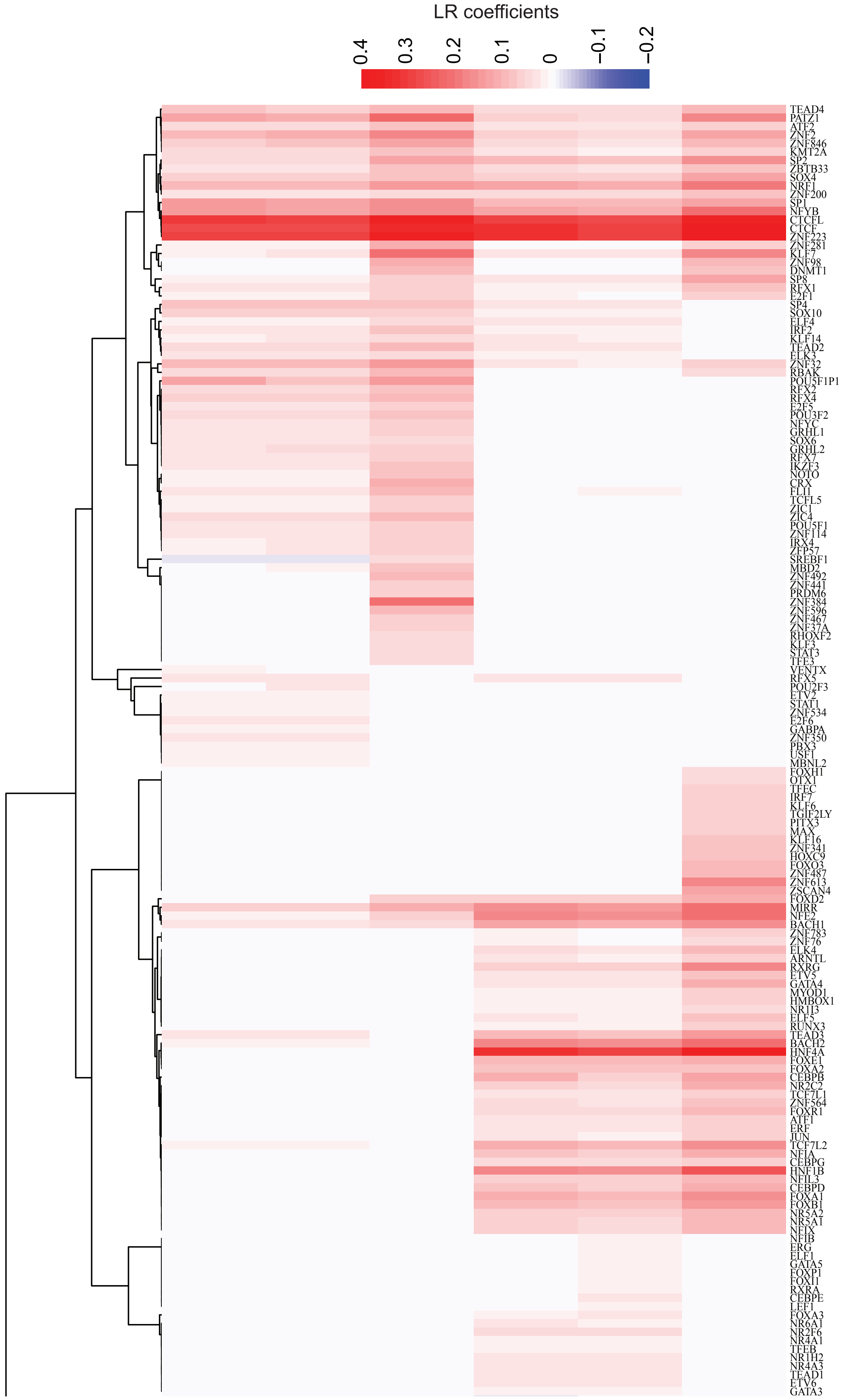
LR coefficients
0.4
0.3
0.2
0.1
0
−0.1
−0.2
TEAD4
PATZ1
ATF2
ZNF2
ZNF846
KMT2A
SP2
ZBTB33
SOX4
NRF1
ZNF200
SP1
NFYB
CTCFL
CTCF
ZNF223
ZNF281
KLF7
ZNF98
DNMT1
SP8
RFX1
E2F1
SP4
SOX10
ELF4
IRF2
KLF14
TEAD2
ELK3
ZNF32
RBAK
POU5F1P1
RFX2
RFX4
E2F5
POU3F2
NFYC
GRHL1
SOX6
GRHL2
RFX7
IKZF3
NOTO
CRX
FLI1
TCFL5
ZIC1
ZIC4
POU5F1
ZNF114
IRX4
ZFP57
SREBF1
MBD2
ZNF492
ZNF441
PRDM6
ZNF384
ZNF596
ZNF467
ZNF37A
RHOXF2
KLF3
STAT3
TFE3
VENTX
RFX5
POU2F3
ETV2
STAT1
ZNF534
E2F6
GABPA
ZNF350
PBX3
USF1
MBNL2
FOXH1
OTX1
TFEC
IRF7
KLF6
TGIF2LY
PITX3
MAX
KLF16
ZNF341
HOXC9
FOXO3
ZNF487
ZNF613
ZSCAN4
FOXD2
MIRR
NFE2
BACH1
ZNF783
ZNF76
ELK4
ARNTL
RXRG
ETV5
GATA4
MYOD1
HMBOX1
NR1I3
ELF5
RUNX3
TEAD3
BACH2
HNF4A
FOXE1
FOXA2
CEBPB
NR2C2
TCF7L1
ZNF564
FOXR1
ATF1
ERF
JUN
TCF7L2
NFIA
CEBPG
HNF1B
NFIL3
CEBPD
FOXA1
FOXB1
NR5A2
NR5A1
NFIX
NFIB
ERG
ELF1
GATA5
FOXP1
FOXI1
RXRA
CEBPE
LEF1
FOXA3
NR6A1
NR2F6
NR4A1
TFEB
NR1H2
NR4A3
TEAD1
ETV6
GATA3

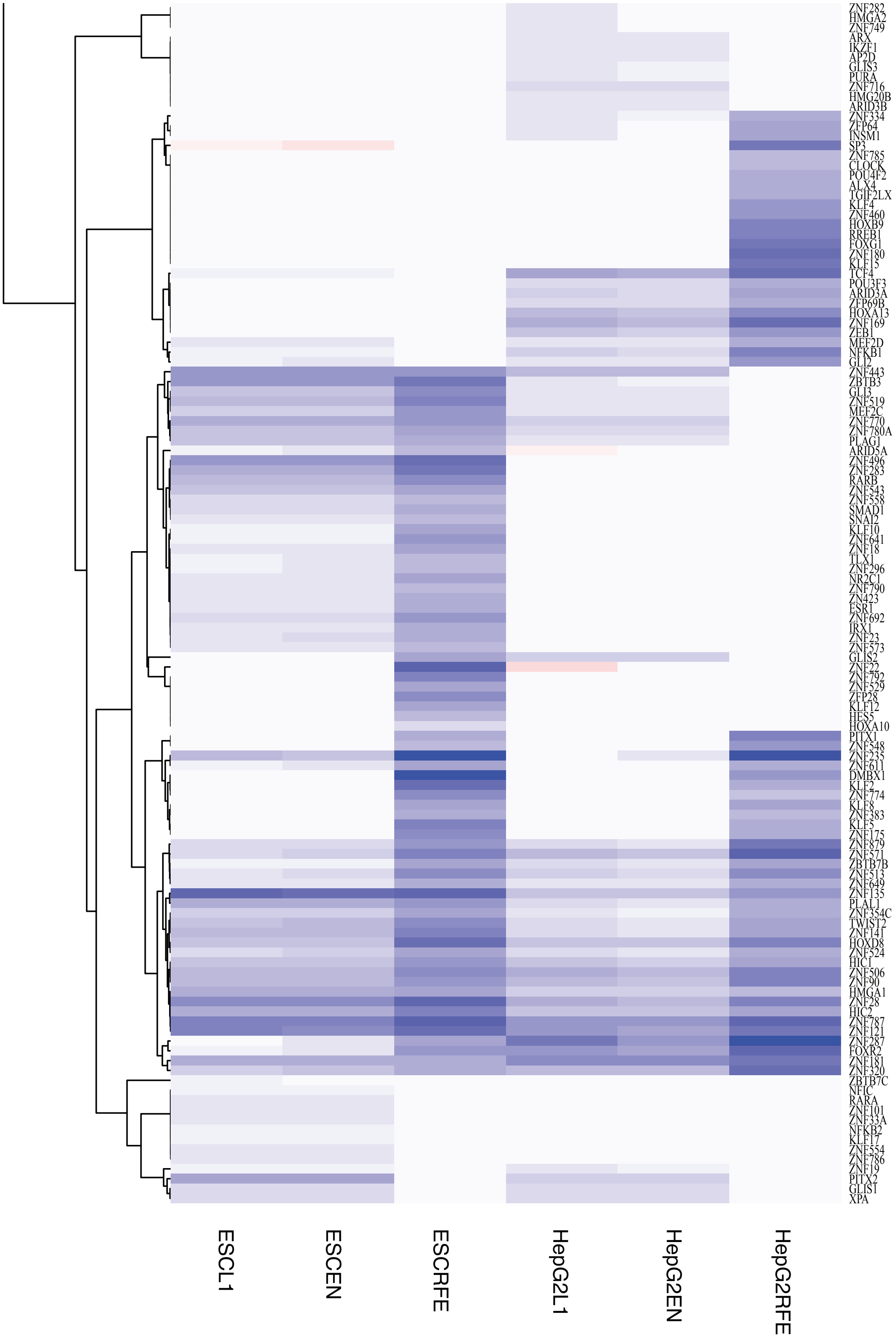

Figure S3: **Heatmap (in Figure 6) with all TF names**

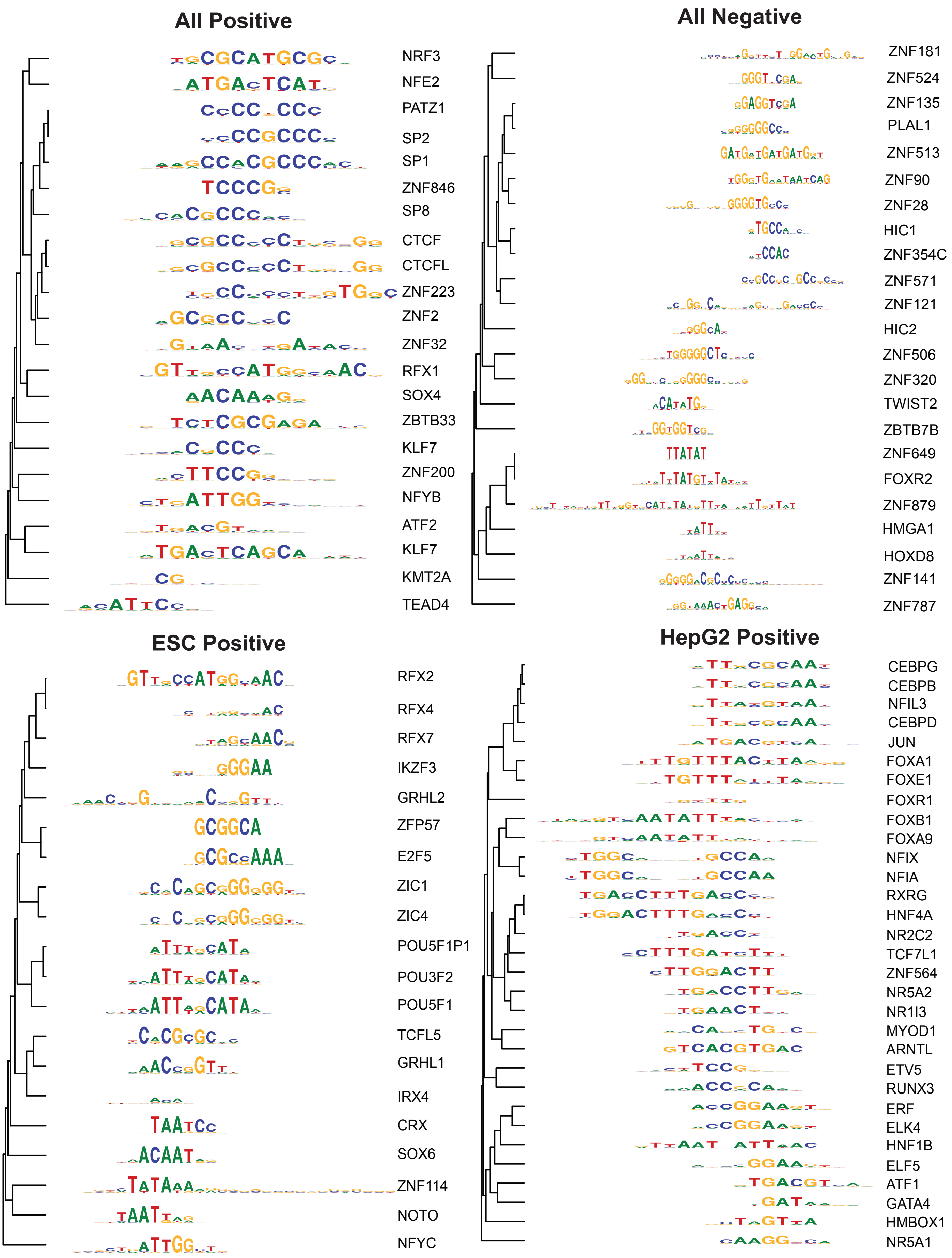

Figure S4: **Motifs of features shown in pullouts in Figure 6**. Motifs were clustered and aligned using tree layout command in MotifStack R package [68].
