# Supplementary material for: Global properties of regulatory sequences are predicted by transcription factor recognition mechanisms
Source: Genome Biol. 2021 Oct 7;22:285. doi: 10.1186/s13059-021-02503-y (PMC8496038; doi:10.1186/s13059-021-02503-y)
Supplement: Supplementary file 6 — Additional file 6. Review history. [file 13059_2021_2503_MOESM6_ESM.docx]

**Review history**

**First round of review**

**Reviewer 1**

"Models of transcription factor binding with or without spacing and orientation constraints predict that regulatory sequences should be fundamentally short, unique, and turn over rapidly." By Patel et al. Manuscript submitted to Genome Biology.

This manuscript collects a number of epigenome mapping datasets, and computationally tests a number of models of how gene regulation and in particular combinatorial TF binding occurs. This type of approach has been greatly improved by the wealth of regulatory mapping of multiple types that has become available in the past decade. The study itself is suitably introduced, well-organized and well-reported. The topic is of broad interest, both theoretical and practical, and is clearly within the remit of GB.

The manuscript concludes that the reported analyses cannot distinguish (or even strongly judge what might be) the operative model that nature is using to generate the regulatory epigenome in mammals. Unfortunately, I think that deeper insight into what mechanisms are operative would enormously improve the impact of this work. Patel of course describe refinements to the likely landscape of regulation and regulatory evolution; these refinements are a respectable, but not spectacular, improvement on our prior understanding.

---Specific points for MS revisions

The title is almost long enough to be the abstract at 25 words.

The results sections could be improved by making their headers more results-focused, instead of: "Length", "Uniqueness", "Master regulators" et cetera, all of which are too terse and somewhat vague.

Table 1 would benefit from a cartoon illustration of all four models to accelerate the reader's understanding. I recommend fusing Table 1 with Figure 1 and elaborating.

Table 1 and 3 were embedded in the text. So I was confused that Table 2 was randomly put near the end. Poor manuscript compilation.

Figure 2. PanelB, traces using different blues are indistinguishable. But these are the most critical.

Figure 3. Overall the figure has relatively low information content. Badly annotated panels.

Figure 4. The most important figure. Requires ruthless simplification of number value reporting for improved clarity. Use of decimalization (e.g. 0.0000000000000000000000000000000009%) in blue and red sectors should be reconsidered, as at that scale becomes pointless. Red example, 3.89% is also probably too many decimal places - should read 3.9 at best. Sounds trivial, but misuse of decimals makes your figure too challenging for a reader to quickly absorb. Table 3 also needlessly challenges the reader with complex numbers.

Remove needless quotes where the concept is not debated
- Repeated use of "master regulators"
-"We then again mutated the "hits" as described above."
-" The notion of a "hierarchy" of TFs, in…"

"and therefore (we assume) they must work together in combination." Please reword.

This phrase is too plodding. "One way to explore sequence properties that dictate regulatory sequence 93 function is to develop and examine computational models, e.g. computer 94 programs that use sequences as inputs and predict class memberships of the 95 sequences, for example regulatory vs. non-regulatory."

Unclear statement. "As gauged by H3K27ac [occupancy of the genome?], only"

**Reviewer 2**

In this manuscript by Patel and Hughes, the authors attempt to address an important, unresolved question in chromatin biology - how do TFs collaborate to encode cell-specific activity of cis-regulatory DNA. Genomics approaches have given rise to various theories into organizing principles of individual regulatory elements. An initial model, typically referred to as the enhanceosome, indicates that the function of an individual cis-element is a combination of TFs that adhere to strict spacing and orientation constraints (SAOCs). In contrast to this rigid model, large-scale chromatin profiling over the last decade has shown the regulatory DNA has (at best) has soft SOACs and individual TF binding sites are subject to a high rate of 'turnover' during evolution (Billboard model). While various lines of genomic evidence have been used to compel each these models, a systematic evaluation of how well each model explains the global sequence and biophysical observation has been lacking. A key reason for this is that modeling the baseline expectations [of observations] is challenging because the genomic sequence and epigenomic observation have strong, yet unknown correlation structure.

Here Patel and Hughes develop a series of clever computational analyses to examine whether models that incorporate spacing and orientation constraints are consistent with global observations of putative regulatory sequences. Interestingly, they find that that models with or without SAOCs are generally compatible with uniqueness, rapidity of evolutionary turnover and their compact size.

Overall, I find this work very compelling, as it represents to my knowledge, the first attempt to directly compared different models of regulatory DNA organization with respect to collective genome-wide observations . For example, the perceived lack of sequence conservation (high evolutionary turnover) of individual TF binding sites has been used as evidence against spacing constraints, however, the baseline expectation has never been tested. While the conclusions of this work are a not surprising (unequal contribution of individual TFs to function; i.e., master TFs important), this represents a straightforward framework to evaluate different models with respect to observed properties of regulatory DNA. Overall, I find this manuscript very strong with only minor comments that would (personally) increase my already substantial enthusiasm.

Comments:

1.     A better description of the how the multimeric motifs were constructed is needed

2.     Lines 217-223: The section pertaining to the whether different models have different implications for size (width) of regulatory DNA: "Thus, while the multimeric motifs cannot be  evaluated here in a predictive format, their length is consistent with regulatory elements typically having a nucleating sequence of fewer than 100 bases." It is unclear to me what this is adding - the multimeric motifs were arbitrarily capped at ~50 bp.

3.     Lines 246-248: "Multimeric motifs are also consistent with the uniqueness of regulatory elements, provided that the number of distinct combinations of TF motifs exceeds the number of regulatory elements in the genome." This sentence was a little confusing - if I understand correctly the authors are saying that if all elements are subject to rigid spacing, constraints and composition the total possible number of multimeric motifs must be greater than the number of total regulatory elements genome-wide (>2 million; DHS and/or H3K27ac). While I agree with this statement in principle, the total of the true number of DHS by unique cellular contexts is likely and order of magnitude lower. In other words, many DHS patterns are highly correlated (Meuleman Nature 2020) and therefore not unique or independent.

4.     Many different approaches have arrived at the 4-5 sites yet it still unclear how this manifests at individual elements. For example, a small number of general (non-lineage specific) TFs (ChIP-seq) data is largely sufficient to qualitatively explain DHS signal (Thurman et all, 2012 - Suppl. Fig. 6). This is consistent with the findings in this manuscript, however because a substantial amount of the regulatory landscape in one cell type is shared with many other cell types it is unclear to what extent the models are learning about how specificity is encoded. It would be good to see the features outputted by the model to see how many motif features are from TFs are expressed in all cell types vs. restricted to H1 ESCs.

5.     Likewise, there is RNA-seq data available through ENCODE for H1 ESCs. When you only include motif models by a TFs that are actually expressed in the LR model (or explicitly including expression in LR model) does the 4-5 TFs still hold?

6.     Regulatory element turnover: How often do mutation create new binding sites that main classification of regulatory DNA in the models? This would have implications in modeling the extent of regulatory DNA repurposing (Vierstra 2015 Science; recently Wang 2020 Science 10.1126/science.aaz3090). More broadly, can this be quantified by building models on additional cell types and looking at the important features unique to each model?

7.     LR model: The LR model is applied uniformly across all DHS, though each DHS might be compositionally dependent on adistinct combination of TFs, though not requiring SAOCs (i.e., interaction model vs independent). Also, the model uses the number of MOODs motif hits as input. It is not clear if the number of TF binding sites is more important than identity. It would be nice to see various implementations of the LR model such as binarizing the input (one motif instance per DHS) and testing different feature selection strategies (e.g., elastic net) to see if the results are significantly different.

**Authors’ response to reviewers**

Reviewer #1:

"Models of transcription factor binding with or without spacing and orientation constraints predict that regulatory sequences should be fundamentally short, unique, and turn over rapidly." By Patel et al. Manuscript submitted to Genome Biology.

“This manuscript collects a number of epigenome mapping datasets, and computationally tests a number of models of how gene regulation and in particular combinatorial TF binding occurs. This type of approach has been greatly improved by the wealth of regulatory mapping of multiple types that has become available in the past decade. The study itself is suitably introduced, well-organized and well-reported. The topic is of broad interest, both theoretical and practical, and is clearly within the remit of GB.”

- - > We appreciate that the referee finds the organization and topic of the paper suitable for GB. We also thank the referee for carefully evaluating the text and figures, for and suggesting the helpful clarifications and modifications below.

“The manuscript concludes that the reported analyses cannot distinguish (or even strongly judge what might be) the operative model that nature is using to generate the regulatory epigenome in mammals. Unfortunately, I think that deeper insight into what mechanisms are operative would enormously improve the impact of this work. Patel of course describe refinements to the likely landscape of regulation and regulatory evolution; these refinements are a respectable, but not spectacular, improvement on our prior understanding.”

- - > We respectfully disagree that the paper is only a refinement of prior understanding. To our knowledge, this work represents the first instance in which properties of regulatory sequences are systematically compared to predominant models about how they operate. Our motivation for packaging the work into a manuscript was motivated by discussions with other investigators at conferences, which revealed that almost no one had made what seem to be fairly simple (albeit tedious) calculations. Previous work in this arena – most notably that of Wunderlich, Mirny, and Sinha – has been very well-cited. Here, we consider numerous aspects of gene regulation that go beyond any previous study, including consideration of highly multimeric motifs.

- - > Nonetheless, we have now augmented the manuscript with additional analyses that we are confident represent the type of deeper insight the referee is referring to. First, in response to Reviewer 2, Comments 4 and 7, we have carefully analyzed the weights of linear models derived from different cell types, which provide strong evidence that many TFs contribute to the identity of regulatory sites in a cell-type-specific manner – thus supporting our estimates made by other approaches. These analyses also highlight now regression models may be instrumental in identifying new cell-type-specific regulators.

- - > Second, we more carefully examine the impact that obligate master regulators would have on generic models of regulatory site specificity, showing (rather than hypothesizing, as previously) that the master regulators should uniformly have the effect of reducing the required complexity of regulatory sites – but also placing additional constraints on the number of TFs that must cooperate in order to achieve the observed properties of regulatory sequences.

- - > We believe that none of the findings described is previously reported in the literature, and all of them are informative towards operative models.

---Specific points for MS revisions

“The title is almost long enough to be the abstract at 25 words.”

- - > We felt that a descriptive title would be helpful, but acknowledge that it is perhaps overly long. We now provide the following (somewhat) abbreviated title: “Global properties of regulatory sequences are predicted by transcription factor recognition mechanisms”

“The results sections could be improved by making their headers more results-focused, instead of: "Length", "Uniqueness", "Master regulators" et cetera, all of which are too terse and somewhat vague.”

- - > The revised paper now has longer and more descriptive headers:

1) “Models are consistent with nucleosome-sized regulatory element lengths”
2) “Models are consistent with regulatory element uniqueness”
3) “Models are compatible with observed frequency of regulatory sites”
4) “Models are consistent with regulatory element turnover”
5) “Models are consistent with a complex regulatory environment that includes master regulators”


“Table 1 would benefit from a cartoon illustration of all four models to accelerate the reader's understanding. I recommend fusing Table 1 with Figure 1 and elaborating.”

- - > We are grateful to the reviewer for this suggestion. Fusing the table and the figure indeed clarifies both.

“Table 1 and 3 were embedded in the text. So I was confused that Table 2 was randomly put near the end. Poor manuscript compilation.”

- - > The formatting requirements of the journal specify that large tables must be appended. In the current version we have simply compressed Table 2 (which is now Table 1) so it fits into the main text. Obviously, we could have done this previously and apologize that it did not occur to us.

“Figure 2. PanelB, traces using different blues are indistinguishable. But these are the most critical.”

- - > The revised Figure 3B now uses a different colour scheme.

“Figure 3. Overall the figure has relatively low information content. Badly annotated panels.”

- - > We have overhauled Figure 4 to reduce the space it occupies, and to improve labelling.

“Figure 4. The most important figure. Requires ruthless simplification of number value reporting for improved clarity. Use of decimalization (e.g. 0.0000000000000000000000000000000009%) in blue and red sectors should be reconsidered, as at that scale becomes pointless. Red example, 3.89% is also probably too many decimal places - should read 3.9 at best. Sounds trivial, but misuse of decimals makes your figure too challenging for a reader to quickly absorb. Table 3 also needlessly challenges the reader with complex numbers.”

- - > Figure 5A (Previously Figure 4A) has been overhauled taking into account the reviewer points.

- Only one decimal place is shown
- Colour scale is changed to highlight (in red) combinations that are within the
observed proportion of the genome that comprises DHSs (0.5% to 3%)

- - > Table 2 (previously Table 3) has also been modified to include only two significant digits, and to use scientific notation instead of programming notation. The numbers do span orders of magnitude – but, their (approximate) values are important to critical points of the manuscript, and thus they are retained.

“Remove needless quotes where the concept is not debated”
- Repeated use of "master regulators"
-"We then again mutated the "hits" as described above."
-" The notion of a "hierarchy" of TFs, in…"

- - > Done

"and therefore (we assume) they must work together in combination." Please reword.

- - > Upon reconsideration, this phrase is unnecessary, and has been removed.

“This phrase is too plodding. "One way to explore sequence properties that dictate regulatory sequence 93 function is to develop and examine computational models, e.g. computer 94 programs that use sequences as inputs and predict class memberships of the 95 sequences, for example regulatory vs. non-regulatory."”

- - > Indeed. Apologies for the bad writing. This sentence has been divided, and now reads: “One way to explore sequence properties that dictate regulatory sequence function is to develop and examine computational models. Computer programs that use sequences as inputs, and predict class memberships of the sequences (for example, regulatory vs. non-regulatory), can be built to incorporate properties such as spacing and orientation of TF binding sites, often using DNA words (k-mers) as a substitute”.

“Unclear statement. "As gauged by H3K27ac [occupancy of the genome?], only"”

- - > This statement now reads “As gauged by H3K27ac occupancy across the genome, only ~40% of liver enhancers are shared between human and macaque, despite these closely-related genomes having a neutral nucleotide substitution rate of only 6%”.

Reviewer #2:

“In this manuscript by Patel and Hughes, the authors attempt to address an important, unresolved question in chromatin biology - how do TFs collaborate to encode cell-specific activity of cis-regulatory DNA. Genomics approaches have given rise to various theories into organizing principles of individual regulatory elements. An initial model, typically referred to as the enhanceosome, indicates that the function of an individual cis-element is a combination of TFs that adhere to strict spacing and orientation constraints (SAOCs). In contrast to this rigid model, large-scale chromatin profiling over the last decade has shown the regulatory DNA has (at best) has soft SOACs and individual TF binding sites are subject to a high rate of 'turnover' during evolution (Billboard model). While various lines of genomic evidence have been used to compel each these models, a systematic evaluation of how well each model explains the global sequence and biophysical observation has been lacking. A key reason for this is that modeling the baseline expectations [of observations] is challenging because the genomic sequence and epigenomic observation have strong, yet unknown correlation structure.”

“Here Patel and Hughes develop a series of clever computational analyses to examine whether models that incorporate spacing and orientation constraints are consistent with global observations of putative regulatory sequences. Interestingly, they find that that models with or without SAOCs are generally compatible with uniqueness, rapidity of evolutionary turnover and their compact size.”

“Overall, I find this work very compelling, as it represents to my knowledge, the first attempt to directly compared different models of regulatory DNA organization with respect to collective genome-wide observations. For example, the perceived lack of sequence conservation (high evolutionary turnover) of individual TF binding sites has been used as evidence against spacing constraints, however, the baseline expectation has never been tested. While the conclusions of this work are a not surprising (unequal contribution of individual TFs to function; i.e., master TFs important), this represents a straightforward framework to evaluate different models with respect to observed properties of regulatory DNA. Overall, I find this manuscript very strong with only minor comments that would (personally) increase my already substantial enthusiasm.”

- - > We are happy that the reviewer had a positive perception of the manuscript.

Comments:

1. “A better description of the how the multimeric motifs were constructed is needed”

- - > We have revised this section of the methods, consulting with two colleagues who agree that the revised version is sufficient to understand and reproduce construction of the multimeric motifs. It now reads as follows: “Multimeric motifs (3 or more motifs) were generated by the following procedure. We first manually annotated dimer motifs (A-B, C-B) from Jolma et al. (2015) to determine if one or both of the monomeric motifs were palindromic, and in what order the TFBSs occurred. We then identified all the possible 3-TF motifs that can be generated accounting for dimers containing a palindromic sequence (3-TF A-B-C motif is only possible if B is palindromic in dimeric motifs A-B and C-B). We then merged dimer motifs into 3-TF motifs by first aligning the two dimers with STAMP (parameters: -cc PCC and -align SWU) [67] to obtain the PFM alignment (manually verifying the correct orientation). At overlapping positions in the alignment, PFM scores in the dimeric motifs were averaged per base, per position to produce the 3-TF motif; scores at non-overlapping positions were unchanged. To generate larger multimeric motifs (4, 5 and 6 TFs) we repeated these steps iteratively by merging the computationally derived 3-TF motifs with dimers to form 4-TF motifs etc.”


2. “Lines 217-223: The section pertaining to the whether different models have different implications for size (width) of regulatory DNA: "Thus, while the multimeric motifs cannot be evaluated here in a predictive format, their length is consistent with regulatory elements typically having a nucleating sequence of fewer than 100 bases." It is unclear to me what this is adding - the multimeric motifs were arbitrarily capped at ~50 bp.”

- - > In fact, we did not arbitrarily cap the multimeric motif lengths. In theory, the multimeric motifs could have been as wide as 150-200 bp, if the original CAP-SELEX experiments (which used 40-mer ligands) had yielded dimeric motifs with larger spacing. Of course, in practice, the spacings are shorter, since the TFs often bind adjacent or overlapping sites. We are uncertain how to modify the paper to clarify this issue but would welcome suggestions.

3. “Lines 246-248: "Multimeric motifs are also consistent with the uniqueness of regulatory elements, provided that the number of distinct combinations of TF motifs exceeds the number of regulatory elements in the genome." This sentence was a little confusing - if I understand correctly the authors are saying that if all elements are subject to rigid spacing, constraints and composition the total possible number of multimeric motifs must be greater than the number of total regulatory elements genome-wide (>2 million; DHS and/or H3K27ac). While I agree with this statement in principle, the total of the true number of DHS by unique cellular contexts is likely and order of magnitude lower. In other words, many DHS patterns are highly correlated (Meuleman Nature 2020) and therefore not unique or independent.”

- - > The referee is correct in what was intended. Because the sentence of concern may be confusing, it has been changed to: “The uniqueness of regulatory elements is also an expected consequence of a model in which multimeric motifs with strict SAOC are predominant, provided that the number of distinct combinations of TF motifs exceeds the number of regulatory elements in the genome.”

- - > It is unclear to us, however, why it is important that the total of the true number of DHS by unique cellular contexts is an order of magnitude lower. The believe the referee intends to convey that any single cell type has only ~100,000 DHS, and not the ~2,000,000 observed in total across cell types – but, the observation that each has a unique sequence is true for the 100,000 as well as the 2,000,000. The fact that the presence and absence many DHS sites are correlated across cell types suggests shared mechanisms, but does not require that the arrangement of sequence features that specify each site will be common among DHSs.

4. “Many different approaches have arrived at the 4-5 sites yet it still unclear how this manifests at individual elements. For example, a small number of general (non-lineage specific) TFs (ChIP-seq) data is largely sufficient to qualitatively explain DHS signal (Thurman et all, 2012 - Suppl. Fig. 6). This is consistent with the findings in this manuscript, however because a substantial amount of the regulatory landscape in one cell type is shared with many other cell types it is unclear to what extent the models are learning about how specificity is encoded. It would be good to see the features outputted by the model to see how many motif features are from TFs are expressed in all cell types vs. restricted to H1 ESCs.”

- - > We are grateful to the referee for this suggestion (and others below) that prompted us to look more carefully at feature weights in different cell types. The paper now contains a new paragraph and figure (the new Figure 6) describing these analyses. Due to computational constraints, instead of all cell types, we examined ESCs and HepG2 for contrast. We tested several different feature selection approaches, which yielded qualitatively similar outcomes. There is a striking correspondence between the feature weights and known biology of the TFs within the cell types. We also find that KRAB-C2H2 zinc finger proteins, an enigmatic class of putative repressors, overwhelmingly constitute a group with negative weights in both cell types. We believe that these observations constitute strong evidence that many TFs contribute to cell type specific regulatory sites – both positively and negatively – and will represent a starting point for future work, as well as a way to identify putative new cell type regulators.

- - > We also examined expression of the genes encoding the corresponding TFs, but did not observe strong correlations with the feature weights, beyond known markers such as OCT4. It is known that mRNA levels do not strictly reflect protein levels, and it is also conceivable that post-translational modifications are in involved. We would also point out that many genes are expressed at low levels and we do not see an obvious way to set a threshold in this analysis. Since this analysis had a negative outcome, and we are hesitant to speculate, we have omitted this observation from the revised paper.

5. “Likewise, there is RNA-seq data available through ENCODE for H1 ESCs. When you only include motif models by a TFs that are actually expressed in the LR model (or explicitly including expression in LR model) does the 4-5 TFs still hold?”

- - > See above regarding expression. We also note that our 4-5 TFs conclusion stems from the multimeric motif analysis, however, not the logistic regression. The manuscript now includes a new section in which we examine how the conclusions of the multimeric motif analysis change in response to differing numbers of expressed TFs (Figure 5C and associated text). Briefly, yes – the 4-5 TFs still holds.

6. “Regulatory element turnover: How often do mutation create new binding sites that main classification of regulatory DNA in the models? This would have implications in modeling the extent of regulatory DNA repurposing (Vierstra 2015 Science; recently Wang 2020 Science 10.1126/science.aaz3090). More broadly, can this be quantified by building models on additional cell types and looking at the important features unique to each model?”

- - > This is a good point – certainly new sites arise - but it is hard to provide a meaningful response, since we can control the rate at which new motifs arise by simply changing the motif score cutoff. We have now added a clarification in this regard to the main text: “We note that the motif score cutoffs reflect the frequency at which motif matches arise in random sequence; thus, by default, such sequences will occur at random. As we do not know the relevant physiological score thresholds, in this analysis we considered loss rates at several motif score cutoffs (presumably reflecting binding strength) and different numbers of cooperating TFs.”

- - > We can also only speculate on repurposing. The Discussion notes that “One possibility is that master regulators could both reduce the complexity and increase adaptability of regulatory sites, by having a single dominant TF binding site and many potential associated cofactors that can change. Master regulators may also be selected for their ability to determine cell type identity in a minimally complex fashion”.

- - > Regarding models built on additional cell types – as noted in our response to point (4) above, we see this approach as a promising means to both identify new cell-type-specific regulators, and to identify regulators important to specific sites. But, such analyses would go beyond the scope of the present work.

7. “LR model: The LR model is applied uniformly across all DHS, though each DHS might be compositionally dependent on a distinct combination of TFs, though not requiring SAOCs (i.e., interaction model vs independent). Also, the model uses the number of MOODs motif hits as input. It is not clear if the number of TF binding sites is more important than identity. It would be nice to see various implementations of the LR model such as binarizing the input (one motif instance per DHS) and testing different feature selection strategies (e.g., elastic net) to see if the results are significantly different.”

- - > Building models involve a number of heuristic decisions and a full grid search is infeasible. Nonetheless, we have performed two of the analysis suggested by the reviewer: Binarizing the input, and testing different feature selection strategies. Binarization did not impact performance, but we note that most of the hits were already 1 or 0, so to us this is not a surprising outcome, and we did not add this finding to the manuscript. Changing the feature selection method also had generally minimal impact, but this does seem a significant outcome, as it shows that the results of the LR analysis are not sensitive to perturbation of the methodology. This analysis is now shown in Figure 6.


List of changes (*have been shown using asterisks*, also highlighted in main text):

1. The title of the paper has been changed and shortened from “Models of transcription factor binding with or without spacing and orientation constraints predict that regulatory sequences should be fundamentally short, unique, and turn over rapidly” to *“Global properties of regulatory sequences are predicted by transcription factor recognition mechanisms”*, as per reviewer 1’s first point.
2. We have moved the former title into the updated the conclusion section of the abstract: *“Models of transcription factor binding with or without spacing and orientation constraints predict that regulatory sequences should be fundamentally short, unique, and turn over rapidly.”* This sentence replaces the sentence “Many observed properties of regulatory sequence appear to be intrinsic to eukaryotic regulatory processes” previously in the conclusion section of the abstract.
3. We have removed the phrase “and therefore (we assume) they must work together in combination” (line 83) based on the suggestion made by reviewer 1 to reword.
4. We have split and updated lines 91-94 in the Introduction to “One way to explore sequence properties that dictate regulatory sequence function is to develop and examine computational models. Computer programs that use sequences as inputs, and predict class memberships of the sequences *(for example, regulatory vs. non-regulatory)*, can be built to incorporate properties such as spacing and orientation of TF binding sites, often using DNA words (k-mers) as a substitute” as per reviewer 1s comments on its phrasing. These sentences previously read “One way to explore sequence properties that dictate regulatory sequence function is to develop and examine computational models, e.g. computer programs that use sequences as inputs and predict class memberships of the sequences, for example regulatory vs. non-regulatory. These programs can potentially learn rules from the vast numbers of regulatory elements and can be built to incorporate properties such as spacing and orientation of TF binding sites, often using DNA words (k-mers) as a substitute.”
5. We modified the sentence in line 96 from “Computational models employing aspects of both models have been successful at classifying regulatory vs non-regulatory sites (e.g. enhancer vs. non-enhancer) much better than random guessing” to “Computational models *with and without SAOC* have been successful at classifying regulatory vs non-regulatory sites (e.g. enhancer vs. non-enhancer) much better than random guessing”, for clarity.
6. We modified the sentence in line 127 “As gauged by H3K27ac, only ~40% of liver enhancers are shared between human and macaque (6% substitution rate)”, due to reviewer 1s concerns regarding its clarity. The sentence now reads *“As gauged by H3K27ac occupancy across the genome, only ~40% of liver enhancers are shared between human and macaque, despite these closely-related genomes having a neutral nucleotide substitution rate of only 6%”*
7. Table 1 (previously table 2) is now embedded in text as per reviewer 1’s suggestions.
8. Line 187 we added the word HepG2 for clarity. The line now reads “A sampling of analyses in another cell type (*HepG2*) yielded similar results.”
9. We added three sentences to the “Dominance of master regulators” row and “Tests” column in Table 1 to account for the additional work we did as a result of reviewer 1 and 2s comments. The two sentences are:
a. *“Examine LR feature weights using multiple feature selection methods.”*
b. *“Poisson estimates of the number of TFs required to specify regulatory sites with and without master regulators.”*
c. *“Multimeric motif hits estimation with and without master regulators”*
10. We updated the headers for the components in the results section and made them more descriptive as per reviewer 1s suggestion. The headers are:
a. “Regulatory Element Length” is now *“Models are consistent with nucleosome-sized regulatory element lengths”*
b. “Regulatory Element Uniqueness” is now *“Models are consistent with regulatory element uniqueness”*
c. “Frequency of regulatory sites” is now *“Models are compatible with observed frequency of regulatory sites”*
d. “Regulatory element turnover” is now *“Models are consistent with regulatory element turnover”*
e. “Master regulators” is now *“Models are consistent with a complex regulatory environment that includes master regulators”*
11. We added the sentence *“Similar results were obtained in the HepG2 cell type as well (Figure S1A)”* to line 216 in the “Models are consistent with nucleosome-sized regulatory element lengths” part of the results section. We extend the length analysis to HepG2 as well as a result of the feature selection suggestion by reviewer 2 (point 7)
12. We expanded the sentence in line 252 for clarity purposes and due to reviewer 2s point 3, by adding the following statement at the start of the sentence: *“The uniqueness of regulatory elements is also an expected consequence of a model in which multimeric motifs with strict SAOC are predominant*, provided that the number of distinct combinations of TF motifs exceeds the number of regulatory elements in the genome.”
13. As per reviewer 1s suggestion, Table 2 (previously Table 3) was corrected to have scientific notation and one decimal place for all values except certain information content values as exact numbers is important for subsequent calculations.
14. We have added two phrases in the sentence starting on line 349 slightly for clarity. “To examine the effects of mutations on SAOC, we identified sequences that have high motif scores for our multimeric motifs from among a very large amount of non-human DNA sequence, in order to obtain a large number of *motif hits* (by the reasoning above, each motif should have only one strong match in the human genome, *resulting in very few examples*).”
15. In the results section titled “Models are consistent with regulatory element turnover”, we added the sentence “*We note that the motif score cutoffs reflect the frequency at which motif matches arise in random sequence; thus, by default, such sequences will occur at random”. As we do not know the relevant physiological score thresholds*, in this analysis we considered loss rates several motif score cutoffs (presumably reflecting binding strength) and different numbers of cooperating TFs.” section of the results.” This clarification was a result of reviewer 2, point 6.
16. We significantly expanded the section of the results pertaining to master regulators, in response to the point made by reviewer 1 deeper operative insight required and taking into account reviewer 2 point 4 and 7

a. We modified line 378 from “We used several approaches to ask whether the models are consistent with the notion of “Master regulators” to “We used several approaches to ask how the models *reflect the existence of master regulators*.
b. We expanded our Poisson calculations (previously Figure 4A) to incorporate projections that incorporate a requirement of master regulators at regulatory sequences (now *Figure 5A, Figure S2, Supplementary table 3 containing calculations)*. We added an entire paragraph on this new analysis in the results section: *“We next considered the case in which a regulatory site requires the existence of a binding site for a hypothetical master regulator (which in this sense could also be considered a selector or pioneer TF), in addition to other specifying factors as above. This notion is suggested by the observation that, in ES cells, half of all regulatory sites contain a binding site for at least one of five master regulators (including CTCF), even though none of the master regulators is sufficient to specify regulatory sites independently (Figure S2). In this analysis, we assumed that the master regulators have the same level of sequence specificity as other TFs. The addition of a requirement for a hypothetical master regulator has the effect of dramatically reducing the number of additional TFs needed to achieve specificity observed in cells (Figure 5A). This outcome holds even allowing for multiple master regulators, each of which can suffice. Thus, while a collective of multiple TFs would still be needed, the master regulator has the effect of reducing the necessary complexity of regulatory sites, because the number of permissible TF combinations drops.”*
c. Line 416 has been modified slightly for clarity “The numbers *of TFs required under plausible scenarios in Figure 5A* are small enough that it should be possible to learn very accurate models, if there are no specific interactions among the TFs.”
d. We also did significant new work on looking at features of the LR model using three different methods (ElasticNet, L1, and RFE) in two different cell types (ESC-H1 and HepG2). This resulted in three new figures *(Figure 6, Figure S1 and Figure S3)*. We added an entire new paragraph on this in the results section: *“To ask what insight could be gained from these models, and whether they are reproducible and consistent with existing knowledge, we examined the features retained in the 150 TF model. We also considered alternative feature reduction methods (ElasticNet and Lasso regression) and another cell type (HepG2). The resulting feature weights for the six models (3 feature selection methods x 2 cell types) are shown in Figure 6. Different feature reduction methods yielded similar overall models for the same cell type, with similar predictive values (Figure S1B). Strikingly, the motif weights strongly reflected known regulatory functions within the corresponding cell types. Features assigned high weights for ES cells included at least six known ES cell regulators, among which were POU5F1 [39], ZFP57 [40], ZNF114 [41], GRHL2 [42], NFYC, and RFX2 [43]. Those with high weights for HepG2 include at least seven TFs with established functions in liver, including HNF4A [44], HNF1B [45], CEBPB [46], FOXA2 [47], NR5A2 [48], GATA4 [49], and NR1I3 [50]. TFs with highest weights in both cell types included known chromatin modulators CTCF [51] and KMT2A [52], promoter-recognition factors Sp1 [53] and NFYB [54], and a panel of TFs with related motifs to CTCF (CTCFL and ZNF223) and SP1 (SP2, SP8, PATZ1) (Figure S3). Intriguingly, 23 motifs are given negative weights in all models; these include 11 KRAB-C2H2 proteins, which are known repressors [55]. It seems unlikely that such a strong correspondence between LR feature weights and independently derived biological properties of the TFs would have been obtained by coincidence; we take this outcome to indicate that the features weights – and the relatively large number of features retained – are biologically meaningful.”*
e. We also incorporated the role of master regulators in multimeric motif hit counts (Previously Figure 4C, now updated *Figure 5C and Supplementary table 3 containing calculations)*. Additionally, we significantly modified the paragraph on this section: *“We also produced corresponding estimates of how many TFs would be needed for multimeric motifs to be consistent with observation in a single cell type, and the impact of incorporating of master regulators. We focused on* four or five TFs cooperating with SAOC in order to specify each regulatory site, as per the analyses above. We calculated how many such combinations *are expected*, and how many regulatory sites would be specified per genome, as a function of the number of TFs expressed, *assuming all of them can contributed to regulatory site formation (Figure 5C). These calculations were then repeated assuming a small number of master regulators (5, 10, or 20) that are obligatory for a regulatory element to function*. In this regime, *with no master regulators*, the number of TFs required to specify the observed number of distinct active regulatory sites per cell type (100,000-200,000) is ~300 (for 4 TF motifs) and ~800 (for 5 TF motifs). *But, if a regulatory site must contain a binding site for at least one of a small number of master regulators, then the number of working combinations of TFs drops precipitously, to a point that most scenarios examined are not feasible. The only workable regimes are those with multimers of 4 TFs, and 700-800 contributing TFs beyond the master regulators. These constraints loosen as the number of master regulators increases (approaching those of the model that does not consider master regulators).”*
f. Lastly, we updated the conclusion of this section by adding the following paragraph: *“Collectively, these outcomes support the notion that regulatory site identity is specified by a relatively large number of factors. The LR models described above do rely most heavily on a relatively small subset of TF motifs, consistent with the notion of master regulators. But, together with the estimates above, they also suggest that many TFs contribute to regulatory site identity, even if they do not significantly influence the overall state of the cell. We also conclude that the existence of master regulators does not exclude models with or without SAOCs, but it does present significant constraints for both models – most dramatically in the case of strict multimeric motifs.”*
17. To reflect the expansion of in the master regulators section of the Results, we expanded on the sentence in Line 506 (Discussion) which now reads “For a model with full SAOC, in the framework described here, only 4- and 5-TF multimeric binding sites yielded feasible numbers, *and only 4-TF sites, coupled with a large number of contributing TFs, are consistent with a model including master regulators across all parameters examined.”*
18. To further underscore the novelty of the manuscript, we added a line to the statement in line 514 “These numbers are also consistent with current estimates (e.g. [56]) and, we believe, conventional wisdom *(but, to our knowledge, it has not been previously demonstrated in this fashion, nor has it been considered by what regime such a system would operate and evolve)”*.
19. We added the following paragraph to the discussion/conclusion section that incorporates our additional analysis on master regulators. *“The results of the LR analysis, however – in which dozens of TFs selected by the model can be easily rationalized on the basis of literature knowledge – suggests that the notion of master regulators is oversimplified, and that the contribution of TFs to regulatory site identity (and, presumably, cell type identity) is instead on a sliding scale. Gene functions with small effect sizes are often difficult to detect in laboratory experiments, and the same is likely true for the effects of TF binding sites. Additionally, the LR model can be used to identify cell type specific regulators.”*
20. The following changes were made to the ML Model section of the methods, to describe the expanded :
a. We added the line: “To scale the hits we used the standard scaler function from Pythons Sci-kit learn library (http://jmlr.csail.mit.edu/papers/v12/pedregosa11a.html).” We scaled to compare feature weights across multiple methods.
b. We added the section on elasticnet and l1 regression *“For feature selection using elasticnet, the following parameters were used in logreg function to obtain ~150 features (penalty: elasticnet, solver: saga, C = 0.0007 and l1_ratio = 0.5). For feature selection using lasso regression, the following parameters were used in the logreg function to obtain ~150 features (penalty: l1, solver: saga, C = 0.00125).”*
21. In response to reviewer 2, point 1, we expanded the methods section “Motif collection and information content calculations” to incorporate a more detailed explanation on multimeric motif generation. The section now reads: *“We first manually annotated dimer motifs (A-B, C-B) from Jolma et al. (2015) to determine if one or both of the monomeric motifs were palindromic, and in what order the TFBSs occurred. We then identified all the possible 3-TF motifs that can be generated accounting for dimers containing a palindromic sequence (3-TF A-B-C motif is only possible if B is palindromic in dimeric motifs A-B and C-B). We then merged dimer motifs into 3-TF motifs by first aligning the two dimers with STAMP (parameters: -cc PCC and -align SWU) [67] to obtain the PFM alignment (manually verifying the correct orientation). At overlapping positions in the alignment, PFM scores in the dimeric motifs were averaged per base, per position to produce the 3-TF motif; scores at non-overlapping positions were unchanged. To generate larger multimeric motifs (4, 5 and 6 TFs) we repeated these steps iteratively by merging the computationally derived 3-TF motifs with dimers to form 4-TF motifs etc.”* The section previously read as: “We first manually annotated dimer motifs (A-B) from Jolma et al. (2015) to determine which were palindromic, and in what order the TFBSs occurred. We then identified all the possible trimers (3-TF motifs) (A-B-C). We then merged dimer motifs into 3-TF motifs by first aligning with STAMP [50] to obtain the PWM alignment (manually verifying the correct orientation), and averaged the PWM scores at the aligned region. These steps were repeated to generate the larger multimeric motifs (4, 5, and 6 TFs).”
22. To reflect the expanded Poisson calculations in the Results, we updated the methods section on the Poisson calculations by adding in the following statement “We then estimated the probability of X hits using the Poisson.Dist(X, Mean, False) function in Excel where the parameters were number of TFs required to specify a regulatory site and the expected mean (i.e. 1/20 * # of active TFs in a cell type and *1/20 * # of active master regulators in cell type). The entire calculations are provided in Supplementary table 3.”*
23. Figure 1 (Merging of previously Table 1 and Figure 1A) is new, based on recommendation by reviewer 1 that this modification would improve the presentation.
24. To accommodate the new Figure 1, the previous Figure 1 B and C are now *Figure 2 A and B*. Figure legend updated was updated to reflect this as well.
25. The previous Figure 2 A,B, and C are now Figure 3 A,B, and C. Figure legend updated was updated to reflect this as well. Additionally, we changed the colour scheme in Figure 2B with regards to reviewer 1s comments.
26. The previous Figure 3 A,B, and C are now Figure 4 A,B, and C. In response to comments of reviewer 1 regarding the organization of this figure, we have made several changes including:
a. Figure title is changed to “*Turnover* of regulatory regions based on simulated mutations at neutral rate” from “Conservation of regulatory regions based on simulated mutations at neutral rate.”
b. Changing the y-axis label from “Proportion conserved” to “Proportion of bases called as regulatory” for Figure 4A.
c. Changing the y-axis label from “Proportion conserved” to “Proportion of DHSs called as positive after mutation” for Figure 4B
d. Changing the y-axis label from “Proportion conserved” to “Proportion of motif hits conserved after mutation” for Figure 4C.
e. Added *“Silhouettes were obtained from PhyloPic”* to end of figure legend.
f. Figure legend was updated to reflect these changes.
27. The previous Figure 4 A,B, and C are now Figure 5 A, B, and C. Figure 5 A and B have been modified to incorporate the new results regarding master regulators. The figure legends have been updated to:
a. For Figure 5A: “a) *Leftmost heatmap* indicates the *cumulative* probability of >X hits (probability of a hit is 1/20 in a 200 bp region) calculated as a Poisson distribution where the parameters are dependent on the number of active TFs in a cell and the numbers of TFs that specify a regulatory site. *Remaining heatmaps show the effect of master regulators in the same framework in which probability in the leftmost heatmap is multiplied by the probability of getting a single master regulator (MR) hit [Poisson(1 MR hit | 1-4 MR available to the cell]. Cells with red shading are close to the expected probability of active regulatory elements in a cell type (i.e. ~0.5-3% of the genome).”*
b. For Figure 5C: “Heatmaps indicating the number of 4-TF and 5-TF multimeric motif hits in the human genome, based on 3% cooperativity rate of dimers *and the presence (5,10,20 MR) and absence of MR. Cells containing values between 30,000 and 200,000 are shaded red corresponding to the typical number of active regulatory elements in a cell type.”*
28. Figure 6 has been added to show LR feature weights as described above in point 14b. Figure 6 legend has been added: *“Figure 6: Exploration of LR feature weights*. Heatmap of feature weights learned by the LR model in two different cell types (ESC-H1 and HepG2) using three different feature selection methods (ElasticNet, L1 and RFE) to obtain ~ top 150 features each. Rows are clustered using hierarchial clustering with uncentered Pearson correlation as the similarity measure and average linkage. Pullouts show four groups of features with the indicated characteristics.”
29. Supplementary Figure 1 has been added, to support the expanded LR analyses describe above: *“Supplementary figure 1: AUROC/AUPRC values for selected classifiers in this study. a) Three ML models (LR, Basset and ls-gkm) were trained (except Basset) and tested on various DHS lengths as input. Positives were DHS sites obtained from the HepG2 cell line. Negatives were random DNA regions of equal length as the positive and matched for GC and repeat element content. b) AUROC/AUPRC values of the LR model trained on DHS sites (200 bp) from ESC-H1 and HepG2 cell types using ~ top 150 features (for each cell type) obtained after feature selection via three different methods (ElasticNet, L1 and RFE).”*
30. Supplementary Figure 2 has been added to show that ESC master regulators + CTCF hits are found in almost 50% of ESC-H1 DHS sites, which is a critical piece of information for new calculations now shown in Figure 5. Figure legend has been added: *“Supplementary figure 2: Motif hits in ESC-H1 DHS sites. a) Number of motif hits for ESC-H1 regulators (Oct4, Sox2, KLF4, and MYC) and CTCF, within ESC-H1 DHS sites. b) Venn diagram indicating overlap of ESC-H1 DHS sites containing motif hits for ESC-H1 regulators (Oct4, Sox2, KLF4, and MYC) and CTCF.”*
31. Supplementary Figure 3 has been added to show the motifs of important features identified in figure 6. Figure legend has been added: *“Supplementary figure 3: Motifs of features shown in pullouts in Figure 6. Motifs were clustered and aligned using tree layout command in MotifStack R package [68].”*
32. Supplementary Table 1 has been updated to include HepG2 data information.
33. Supplementary Table 2 has been added to include CIS-BP Ids for the motifs used in LR model.
34. Supplementary Table 3 has been added to show the workbook containing Poisson calculations and Multimeric Motif Hit calculations in Table 2 and Figure 5C.

**Second round of review**

**Reviewer 2**

The authors have sufficiently addressed my concerns in this revision.
